# Multi-dimensional cell-free DNA-based liquid biopsy for sensitive early detection of gastric cancer

**DOI:** 10.1186/s13073-024-01352-1

**Published:** 2024-06-07

**Authors:** Pengfei Yu, Ping Chen, Min Wu, Guangyu Ding, Hua Bao, Yian Du, Zhiyuan Xu, Litao Yang, Jingquan Fang, Xingmao Huang, Qian Lai, Jia Wei, Junrong Yan, Shanshan Yang, Peng He, Xue Wu, Yang Shao, Dan Su, Xiangdong Cheng

**Affiliations:** 1 Department of Gastric Surgery, Zhejiang Cancer Hospital, Hangzhou Institute of Medicine (HIM), Chinese Academy of Sciences, Hangzhou, Zhejiang 310022 China; 2 Department of Gastrointestinal Surgery, Ningbo No.2 Hospital, Ningbo, Zhejiang 315010 China; 3 Geneseeq Research Institute, Nanjing Geneseeq Technology Inc., Nanjing, Jiangsu 210032 China; 4 Department of Pathology, Zhejiang Cancer Hospital, Hangzhou Institute of Medicine (HIM), Chinese Academy of Sciences, Hangzhou, Zhejiang 310022 China; 5 School of Public Health, Nanjing Medical University, Nanjing, Jiangsu 211166 China

**Keywords:** Liquid biopsy, Cell-free DNA, Early detection, Gastric cancer, Fragmentomics

## Abstract

**Background:**

Gastric cancer is the fifth most common cancer type. Most patients are diagnosed at advanced stages with poor prognosis. A non-invasive assay for the detection of early-stage gastric cancer is highly desirable for reducing associated mortality.

**Methods:**

We collected a prospective study cohort of 110 stage I–II gastric cancer patients and 139 non-cancer individuals. We performed whole-genome sequencing with plasma samples and profiled four types of cell-free DNA (cfDNA) characteristics, fragment size pattern, copy number variation, nucleosome coverage pattern, and single nucleotide substitution. With these differential profiles, we developed an ensemble model to detect gastric cancer signals. Further, we validated the assay in an in-house first validation cohort of 73 gastric cancer patients and 94 non-cancer individuals and an independent second validation cohort of 47 gastric cancer patients and 49 non-cancer individuals. Additionally, we evaluated the assay in a hypothetical 100,000 screening population by Monte Carlo simulation.

**Results:**

Our cfDNA-based assay could distinguish early-stage gastric cancer from non-cancer at an AUROC of 0.962 (95% CI: 0.942–0.982) in the study cohort, 0.972 (95% CI: 0.953–0.992) in the first validation cohort and 0.937 (95% CI: 0.890–0.983) in the second validation cohort. The model reached a specificity of 92.1% (128/139) and a sensitivity of 88.2% (97/110) in the study cohort. In the first validation cohort, 91.5% (86/94) of non-cancer individuals and 91.8% (67/73) of gastric cancer patients were correctly identified. In the second validation cohort, 89.8% (44/49) of non-cancer individuals and 87.2% (41/47) of gastric cancer patients were accurately classified.

**Conclusions:**

We introduced a liquid biopsy assay using multiple dimensions of cfDNA characteristics that could accurately identify early-stage gastric cancer from non-cancerous conditions. As a cost-effective non-invasive approach, it may provide population-wide benefits for the early detection of gastric cancer.

**Trial registration:**

This study was registered on ClinicalTrials.gov under the identifier NCT05269056 on March 7, 2022.

**Supplementary Information:**

The online version contains supplementary material available at 10.1186/s13073-024-01352-1.

## Background

Gastric cancer is the third leading cause of cancer-associated mortality worldwide [1]. Patients diagnosed at early stages have a favorable prognosis, highlighting the clinical significance of the early detection for reducing gastric cancer-associated mortality. The current standard screening modality, endoscopic surveillance accompanied by pathological examination of tissue biopsies, is limited to high-risk populations due to its invasiveness and high cost. Therefore, as a cost-effective and non-invasive assay, cell-free DNA (cfDNA)-based liquid biopsy may be more suitable for regular screening and holds the potential for detecting early-stage diseases.

Multiple aspects of cfDNA characteristics have been shown to harbor signals of circulating tumor DNA (ctDNA) in various cancer types. For example, cancer patients tend to have more fragmented circulating DNA with shorter lengths [2]. Therefore, a left-shifted cfDNA fragment size distribution may indicate the presence of ctDNA-shedding tumors. The cfDNA fragment coverage could be altered by epigenetic regulations in tissues [3]. Consequently, the altered cfDNA fragment coverage patterns at transcription start sites may reflect aberrant gene expression levels in cancers [4]. Additionally, the copy number variations inferred from cfDNA data also have been shown to correspond with those in tissues [5]. Abnormal copy number losses and gains in cfDNA may be contributed by tumor cells. More recently, mutational signatures in cfDNA have been found to be sensitive enough to detect cancer [6]. Despite promising evidence for these cfDNA features as potential biomarkers to identify cancers, none have been previously evaluated in an early-stage gastric cancer study.

In this study, we prospectively collected a cohort of 249 participants, among which 110 were pathologically confirmed with stage I–II gastric cancer and 139 with non-cancerous conditions. A plasma sample was collected from each patient before standard screening modalities for gastric cancer. We performed whole-genome sequencing with plasma samples and investigated four types of cfDNA profiles, fragment size pattern (FSP), copy number variation (CNV), nucleosome coverage pattern (NCP), and single nucleotide substitution (SNS). We depicted the differences between gastric cancer and non-cancer groups and developed an ensemble model using all four types of cfDNA profiles to infer the probability of cancer for each sample. We later collected two validation cohorts to validate the model’s capability in identifying early-stage gastric cancer. The first validation cohort was from the same center, but temporally separated from the study cohort, including 73 stage I–II gastric cancer patients and 94 non-cancer individuals. The second was from an independent center, consisting of 47 stage I–II gastric cancer patients and 49 non-cancer individuals. Through these efforts, we hope to provide a cfDNA-based liquid biopsy framework that could be implemented in the clinic for the early detection of gastric cancer.

## Methods

### Study population and samples

The study cohort consisted of 110 patients with pathologically confirmed gastric cancer and 139 participants with non-cancerous conditions enrolled at Zhejiang Cancer Hospital (Hangzhou, China) between October 1^st^, 2021, and May 30^th^, 2022. Following the completion of training cohort enrollment, a separate validation cohort comprising 73 gastric cancer patients and 94 non-cancer individuals was collected at the same site from June 1^st^, 2022, to October 31^st^, 2022 (Additional file 1: Fig. S1). All participants provided written informed consent. Each participant had a peripheral blood sample (~ 10 ml) collected before other screening tests for gastric cancer, including gastroscopy, CT scans, and blood tests of tumor markers. Plasma was isolated from blood samples within 4 h. cfDNA was then extracted within 72 h and stored at – 80 ℃ for further cfDNA sequencing. All processes were conducted in Zhejiang Cancer Hospital (Hangzhou, China). Both cohorts followed the same protocols for participant enrollment and sample preparations, except that 31 samples collected between September 2022 and October 2022 were processed with a new automated liquid handling platform.

A second validation cohort consisting of cfDNA samples of 47 gastric cancer patients and 49 non-cancer individuals was retrospectively collected from an independent center, Ningbo No.2 Hospital (Ningbo, China). Cases in the Biobank were first filtered using the same inclusion and exclusion criteria as the study cohort. Stratified random selection was then performed to ensure that the demographic distributions (age, sex, postsurgical stage, tumor location, Lauren’s classification, and non-cancerous disease status) of this validation cohort mirrored those of the study cohort. Sample preparations followed the same protocols as the study cohort.

The study was approved by the institutional review board of Zhejiang Cancer Hospital and performed in accordance with the principles of the Declaration of Helsinki.

### Cohort inclusion and exclusion criteria

Participants meeting the following criteria were considered eligible for enrollment: (1) individuals aged between 18 and 85 years old; (2) individuals without a prior history of cancer; (3) individuals with a plasma sample that was collected before initial screening tests and passed QC; and (4) individuals who provided informed consent. Eligible individuals were assigned to the appropriate group if they further met the following criteria: (1) gastric cancer group: individuals who were pathologically confirmed with gastric adenocarcinomas by biopsies without concomitant malignancies and received curative surgeries for gastric cancer; and (2) non-cancer group: individuals who showed no signs of cancers based on routine physical examinations, including blood tests, ultrasound and CT, and no sign of gastric cancer from gastric cancer-specific screening tests. The size of the non-cancer group was controlled to match the gastric cancer group.

Participants meeting any of the following criteria were excluded from the gastric cancer group: (1) individuals who were pathologically diagnosed with stage III/IV gastric cancer; (2) individuals who withdrew informed consent; and (3) individuals whose sequencing data failed QC.

Participants meeting any of the following criteria were excluded from the non-cancer group: (1) individuals with significant chronic diseases of other systems, such as severe cardiovascular diseases, uncontrolled diabetes, hypertension, and infectious diseases; (2) Individuals who had abnormal results of tumor marker (CEA, CA19-9, CA125, PSA, AFP, etc.) examinations within the past year; (3) individuals who withdrew informed consent; and (4) individuals whose sequencing data failed QC.

### Sample and sequencing library preparations

Peripheral blood samples were first centrifuged at 16,000 × at 4 °C for 10 min to aliquot plasma and serum separately. Circulating cell-free DNA (cfDNA) was isolated from 2 to 4 mL of plasma using the QIAamp Circulating Nucleic Acid Kit (Qiagen), following the manufacturer’s protocols. The concentrations of isolated cfDNA were examined using Qubit dsDNA HS Assay Kit (Thermo Fisher Scientific). cfDNA libraries for whole-genome sequencing (WGS) were prepared using the KAPA Hyper Prep Kit (KAPA Biosystems) according to the manufacturer’s protocol. In brief, 5–10 ng of cfDNA per sample was subjected to end-repairing, A-tailing, and ligation with adapters sequentially. The Hamilton Microlab STAR automated liquid handling platform (Hamilton Company) (hereafter denoted as Platform 1) was used for the automated pipeline operations. For 31 samples in the first validation cohort that were collected between September 2022 and October 2022, the Beckman Biomek i7 automated liquid handling platform (Beckman) (hereafter denoted as Platform 2) was used. The libraries were quantified using the KAPA SYBR FAST qPCR Master Mix (KAPA Biosystems).

### Low-coverage whole-genome sequencing and alignment

Whole-genome libraries were sequenced using 100-bp paired-end runs on the NovaSeq platforms (Illumina) at 8X coverage per genome according to the manufacturer’s instructions. Trimmomatic [7] was used for FASTQ file quality control. The Picard toolkit was used to remove PCR duplicates (http://broadinstitute.github.io/picard/). Qualified reads were then aligned to the human reference genome (GRCh37/UCSC hg19) using the Burrows–Wheeler Aligner (BWA) [8]. To mitigate potential biases introduced by varying sequencing depths among samples, all plasma samples were down-sampled to a uniform coverage of 5 × .

### Whole-genome sequencing features

#### Fragment size pattern

The extraction of fragment size features was adapted from the method introduced by D. Mathios et al. [9]. A non-parametric method for fragment-level adjustment for GC content and library size was first performed. We tiled the reference genome into non-overlapping 5-Mb bins and preserved a total of 541 bins with an average GC content ≥ 0.3 and an average mappability ≥ 0.9. We then computed the adjusted number of short (100–150 bp) and long (151–220 bp) fragments in each bin and standardized the numbers to have mean zero and unit standard deviation across all bins.

#### Copy number variation

The ichorCNA tool was used to profile copy number variations as described by Wan et al. [10]. The reference genome was tiled into non-overlapping 1-Mb bins, totaling 2475 bins. The depth of each bin was used to compare against the software baseline and compute the log2 ratio.

#### Nucleosome coverage pattern

The cfDNA fragmentation coverage patterns at transcription start sites (TSSs) were reported to be associated with epigenetic regulation. Therefore, cfDNA coverage patterns at certain TSS regions may aid the detection of cancer. The selection of TSSs and profiling of related nucleosome coverage patterns followed the method described by Doebley et al. [4]. We used the GTRD database (v 21.12, https://gtrd.biouml.org/downloads/21.12/chip-seq/) [11] to select transcription factors (TFs) with more than 10,000 highly mappable sites. TFs falling beyond a list of TFs with known binding sites in the CIS-BP database (v2.00, http://cisbp.ccbr.utoronto.ca/bulk.php) [12] were excluded. A total of 854 TFs were finally selected. For each TF, we selected 10,000 mappable sites with the highest peak counts for the analyses of coverage patterns.

To profile the nucleosome coverage pattern of a TF, we first extracted all reads of lengths between 100 and 220 bp in a window (− 5 kbp to + 5 kbp) around each binding site of the TF. For each read, we assigned a weight of the reciprocal of its GC bias. For each site, we split it into 15-bp bins and summed the weights of all reads whose midpoints fall within each bin, generating a GC-corrected midpoint coverage profile. For each TF, we averaged the midpoint coverage profiles of all its binding sites and normalized the 10-kbp window coverages to a mean of 1. The resulting coverage curve along the 10 kb window was then smoothed using a Savitzky-Golay filter with a window length of 150 bp and a polynomial order of 3 to generate the final coverage profile of the TF.

We extracted three features to quantify the coverage profile of each of the 854 selected TFs: (1) the mean coverage of the region flanking 1 kbp upstream and 1 kbp downstream of the center; (2) the coverage at the center; and (3) the amplitude of coverage peaks surrounding the center by a fast Fourier transform.

#### Single nucleotide substitution signature

The single nucleotide substitution (SNS) signatures were derived from six types: C > A, C > G, C > T, T > A, T > C, and T > G. Taking into account one upstream and one downstream adjacent base, a total of 6 × 4 × 4 = 96 combinations were involved. The relative abundance of each combination was calculated and denoted as the signature. The processes were adapted from the method developed by Wan et al. [6].

Raw FASTQ files were first trimmed and aligned to the hg19 genome, and duplicate reads were removed before sorting and indexing into BAM files by SAMtools (version 1.9) [13]. The average sequencing depth was calculated from the BAM files. Reads of alternative alignment or template length > 300 bp were excluded. Repeat or low-complexity regions were masked before further analyses. GC bias metrics were calculated using Picard (version 2.19.0, http://broadinstitute.github.io/picard/) with a bin size of 400 bp, and the average GC profile, which were later used to normalize the mutation counts by the GC content of each read, were determined by LOESS smoothing method. Paired reads with only 1 mismatch with reference genome were retained, and BCFtools (version 1.9) [14] mpileup were used along with multiallelic-caller to call SNS mutations from reads that met the following criteria: base quality ≥ 30 and mapping quality ≥ 60. InDels were not included. The identified mutations were annotated by ANNOVAR (version 2016–04-25) using RefSeq and dbSNP [15]. SNP sites were then filtered out from the SNS variants to minimize the influence of germline mutations. Clonal hematopoiesis of indeterminate potential (CHIP) mutations was filtered out using an in-house list of frequent CHIP mutations generated from a normal pool of 1000 healthy individuals. The filtered SNS variants were categorized into the 96 types as described above. Counts of each type were calculated and normalized based on the mean sequencing depth.

To further reduce the effects of potential noises from various sources in healthy populations, a baseline control was built from an internal sample pool of 300 healthy individuals. The 96-SNS signatures of each healthy control were generated through the same processes as described above. The baseline was set as the mean value over the 300 controls for each SNS signature. The final SNS signatures of a test sample were calculated as the raw signature values subtracting the baseline values.

### Machine learning and cross-validation analyses

We built a two-layer machine learning classifier for the malignant nodules and benign ones. The first-layer module took one type of the features (FSP, NCP, CNV, SNS) as the input. For each feature type, the module iterated through four algorithms, elastic-net logistic regression (GLM), extreme gradient boosting (XGBoost), random forest (RF), and neural network (NN). Hyperparameter tuning was conducted using random search across a list of candidate values for each algorithm. These processes resulted in the generation of over 200 models. From this pool, we identified five algorithm-hyperparameter combinations that yielded the highest classification area under the receiver operator characteristic curve (AUROC) metrics, without consideration for the underlying algorithms. These top-performing combinations were selected as the first-layer 20 base models (4 feature types X 5 best algorithm-hyperparameter combinations = 20 base models) for later second-layer stacking.

The second-layer module stacked the 20 base models with algorithm iteration (GLM, XGBoost, and RF) and hyperparameter tuning, also generating over 100 candidate algorithm-hyperparameter combinations. From these combinations, five stacked models with the best AUROC metrics were saved. The prediction scores from these five models were averaged to generate the final outputs of the classifier. In the study cohort, the classifier was evaluated through fivefold cross-validations. For the validation cohort, the models were fitted using the full study cohort and then used to predict the validation samples.

### Rank-based variable importance

The default variable importance from different machine learning algorithms had different magnitudes and thus could not be merged. Our assay stacked models of multiple algorithms for each feature type. To evaluate the relative importance of feature variables across these models, we used their ranks in the default variable importance in each base model, which were of no magnitude. For each base model, we assigned a rank score for 25 variables of the highest default importance from 25 to 1. All variables ranked beyond 25th would be assigned a uniform score of 1. Each base model was assigned a weight score based on its rank in the second-layer model. For the ensemble model, 20 base models were stacked. The weight scores thus ranged from 20 for the most important base model to 1 for the least. The rank scores of a variable in different base models were then averaged with the weights. Within each feature type, the relative rank-based importance of a variable was calculated as its weighted average rank score, standardized to the max score of this feature type. This ensures that the most important variable has a relative importance value of 1.

### Simulation for estimating assay performance in a screening population

To assess and compare the performance of gastroscopy and our cfDNA-based assay in a hypothetical screening population of 100,000 individuals, we used Monte Carlo simulations to capture the uncertainty of parameters such as the sensitivity, specificity, participant compliance rate, and gastric cancer prevalence. These parameters were sampled with prior distributions centered on empirical estimates obtained from published large-scale studies and this study. The method was adapted from a published study by Mathios et al. [16].

Hamashima et al. reported a sensitivity of 88.6% (95% CI: 69.8–97.6%) and a specificity of 85.1% (95% CI: 84.3–85.9%) for gastroscopy from 7388 screenings [17]. With the R package epiR (version 1.0–14, https://fvas.unimelb.edu.au/research/groups/veterinary-epidemiology-melbourne), we assumed that the prior distributions for the sensitivity and specificity of gastroscopy were Beta(22,3.7) and Beta(6000,1051), respectively. From the performance metrics of our cfDNA-based assay in the validation cohort (sensitivity: 91.8%, 95% CI: 83.2–96.2%; specificity: 94.5%, 95% CI: 84.1–95.6%), we assumed the respective prior distributions as Beta(67,6) and Beta(86,8).

Zeng et al. reported a populational compliance rate of 43.8% for gastroscopic screenings from a multi-center trial of 230,583 subjects [18]. Therefore, the prior model for gastroscopy compliance rate was Beta(43.8, 56.2). For non-invasive cfDNA-based tests, the compliance rate was conservatively estimated at 80.0%, leading to an assumed prior distribution of Beta(80.0, 20.0). The study also provided a populational gastric cancer prevalence of 0.8%. Thus, for a given screening population (*N*), the number of gastric cancer cases may be estimated with a distribution of Binomial(0.8%, *N*).

With all these priors, we conducted the Monte Carlo simulation as follows:

1. Sampled the probabilities of compliance rates (C) from the prior beta distributions described above:
  *C*_endo_ ~ Beta(43.8, 56.2)
  *C*_ctDNA_ ~ Beta(80, 20)
2. Simulated the number of individuals that completed the tests (N) in the 100,000 screening cohort from binomial distributions with the probabilities of compliance rates (C):
  *N*_endo_ ~ Binomial(C_endo_, 100,000)
  *N*_ctDNA_ ~ Binomial(C_ctDNA_, 100,000)
3. Simulated the number of gastric cancer cases (S) in the participants from binomial distributions with the populational gastric cancer prevalence (0.8%):
  *S*_endo_ ~ Binomial(0.008, *N*_endo_)
  *S*_ctDNA_ ~ Binomial(0.008, *N*_ctDNA_)
4. Sampled the sensitivity (SE) and specificity (SP) from the prior beta distributions described above:
  SE_endo_ ~ Beta(22, 3.7)
  SE_ctDNA_ ~ Beta(67, 6)
  SP_endo_ ~ Beta(6000, 1051)
  SP_ctDNA_ ~ Beta(86, 8)
5. Simulated the number of true positive (TP) and true negative (TP) incidences from binomial distributions:
  TP_endo_ ~ Binomial(SE_endo_, *S*_endo_)
  TP_ctDNA_ ~ Binomial(SE_ctDNA_, *S*_ctDNA_)
  TN_endo_ ~ Binomial(SP_endo_, *N*_endo_ − *S*_endo_)
  TN_ctDNA_ ~ Binomial(SP_ctDNA_, *N*_ctDNA_ − *S*_ctDNA_)
6. With true positive incidences (TP), true negative incidences (TN), total gastric cancer cases (S), and total testing cases (N), we were able to calculate the negative prediction value NPV = TN/(TN + S − TP) and false negative rate FNR = (S − TP)/N.

We ran the simulation for 10,000 iterations and obtained the posterior distributions of the performance metrics, which allowed for comparisons between the two methodologies in a large hypothetical population with stochastic effects considered.

### Gastric tumor location

The locations of gastric tumors were divided into three groups: proximal, middle, and distal. The proximal group included the cardia and fundus zones. The middle group included the stomach body regions. The distal group included the incisura angularis and pyloric antrum zones.

### Outcomes and timelines

This observatory study was registered on ClinicalTrials.gov under the identifier NCT05269056 on March 7, 2022. The primary outcome measure of the registered trial is the AUROC metric for differentiating stage I/II gastric cancer patients and non-cancer individuals using the cfDNA-based assay. The secondary outcome measures are the sensitivity and specificity of the assay.

The enrollment dates of the first and last participants in the study cohort were October 1^st^, 2021, and May 30^th^, 2022, respectively. The enrollment dates of the first and last participants in the first validation cohort were June 3^rd^, 2022, and October 31^st^, 2022, respectively. The model construction and assessment of results in the study cohort started on July 1^st^, 2022, after the completion of data collection of the study cohort. The assessment of results in the validation cohort started on December 1^st^, 2022.

### Statistical analysis

The comparison of continuous numeric data was done using the Wilcoxon test. The comparison of proportions between groups was done using Fisher’s exact test. The trend of continuous numeric data across ordered groups was assessed using the Jonckheere trend test. A two-sided *P* value of less than 0.05 was considered significant for all tests unless otherwise indicated. All statistical analyses were performed in R (v4.0.2).

## Results

### Cohort overview

**Fig. 1:**
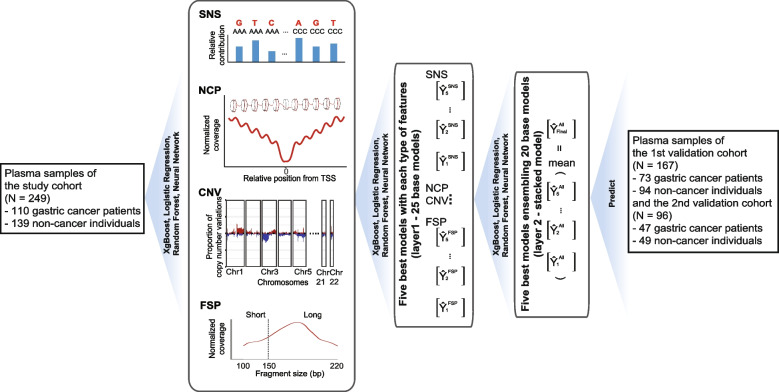
Overview of study and cfDNA profiles. Flowchart illustrating the cohort inclusion, model construction and model validation

This study enrolled a training cohort of 249 individuals at Zhejiang Cancer Hospital, China, between October 1^st^, 2021, and May 30^th^, 2022. Over two fifths (44.2%, 110/249) of them were pathologically confirmed with gastric cancer. The remaining 139 (55.8%, 139/249) had normal results of endoscopy-based screenings for gastric cancer (Fig. 1). Patients with gastric cancer on average were 0.8 years older than non-cancer individuals (58.1 ± 11.0 vs. 57.3 ± 10.3). Most participants were male, making up 56.4% (62/110) and 59.0% (82/139) of the gastric cancer and non-cancer group, respectively. The majority of gastric cancer patients were at stage I (77.3%, 85/110). Gastric tumors at distal locations such as the pylorus and antrum were easier to detect via gastroscopy, which comprised 60.9% (67/110) of the cancer group. On the contrary, only 10.9% (12/110) were composed of proximal tumors. More intestinal types of gastric cancer (45.4%, 50/110) by Lauren’s criteria were included than the diffuse (31.8%, 35/110) and mixed (22.7%, 25/110) types. Among non-cancer participants, about 10.1% (14/139) had gastric diseases (Table 1; Additional file 2: Table S1).

**Table 1:** Participant demography

Cohort	**Study (*n* = 249)**		**Validation (*n* = 167)**		**2nd Validation (*n* = 96)**	
	Cancer (*n* = 110)	Non-cancer (*n* = 139)	Cancer (*n* = 73)	Non-cancer (*n* = 94)	Cancer (*n* = 47)	Non-cancer (*n* = 49)
**Age**						
Median (min–max)	58.0 (26.0–80.0)	58.0 (27.0–85.0)	60.0 (30.0–76.0)	59.0 (27.0–84.0)	57.0 (43.0–82.0)	58.0 (40.0–74.0)
Mean ± SD	58.1 ± 11.0	57.3 ± 10.3	59.3 ± 10.4	57.9 ± 10.9	57.2 ± 8.6	58.3 ± 7.9
**Sex**						
Female	48 (43.6%)	57 (41.0%)	24 (32.9%)	40 (42.5%)	23 (48.9%)	23 (46.9%)
Male	62 (56.4%)	82 (59.0%)	49 (67.1%)	54 (57.4%)	24 (51.1%)	26 (53.1%)
**Postsurgical stage**						
I	85 (77.3%)	/	56 (76.7%)	/	35 (74.5%)	/
II	25 (22.7%)	/	17 (23.3%)	/	12 (25.5%)	/
**T stage**						
1	85 (77.3%)	/	48 (65.8%)	/	41 (87.2%)	/
2	13 (11.8%)	/	16 (21.9%)	/	4 (8.5%)	/
3	10 (9.1%)	/	4 (5.5%)	/	2 (4.3%)	/
4	2 (1.8%)	/	5 (6.8%)	/	0 (0.0%)	/
**N stage**						
0	86 (78.2%)	/	59 (80.8%)	/	37 (78.7%)	/
1	16 (14.5%)	/	10 (13.7%)	/	8 (17.0%)	/
2	7 (6.4%)	/	1 (1.4%)	/	2 (4.3%)	/
3	1 (0.9%)	/	3 (4.1%)	/	0 (0.0%)	/
**Location**						
Proximal	12 (10.9%)	/	7 (9.6%)	/	6 (12.8%)	/
Middle	31 (28.2%)	/	20 (27.4%)	/	12 (25.5%)	/
Distal	67 (60.9%)	/	46 (63.0%)	/	29 (61.7%)	/
**Lauren classification**						
Diffuse	35 (31.8%)	/	30 (41.1%)	/	14 (29.8%)	/
Intestinal	50 (45.4%)	/	28 (38.4%)	/	16 (34.0%)	/
Mixed	25 (22.7%)	/	15 (20.5%)	/	17 (36.2%)	/
**Disease**						
Healthy	/	125 (89.9%)	/	85 (90.4%)	/	44 (89.8%)
Disease	/	14 (10.1%)	/	9 (9.6%)	/	5 (10.2%)

Clinicopathological characteristics of participants in the study and validation cohorts

A validation cohort consisting of 73 gastric cancer patients (43.7%) and 94 non-cancer individuals (56.3%) was separately collected at the same site following the completion of training cohort enrollment, between June 1^st^, 2022, and October 31^st^, 2022. The mean age of gastric cancer patients (59.3 ± 10.4) was 1.4 years greater than that of non-cancer participants (57.9 ± 10.9). 67.1% (49/73) of gastric cancer patients and 57.4% (54/94) of non-cancer individuals were male. Similarly to the training cohort, over three quarters (76.7%, 56/73) of gastric cancer were stage I diseases. Similarly, the proportions of gastric tumors at distal and middle regions were also 63.0% (46/73) and 27.4% (20/73), respectively. A difference from the training cohort was that patients with diffuse-type gastric cancer (41.1%, 30/73) were slightly more than those with the intestinal type (38.4%, 28/73). As for the control group, a small number of non-cancerous gastric diseases were also included (9.6%, 9/94) (Table 1; Additional file 2: Table S1).

A second independent validation cohort consisting of 47 gastric cancer patients (49.0%) and 49 non-cancer individuals (51.0%) was retrospectively collected from Ningbo No.2 Hospital. The mean age of gastric cancer patients (57.2 ± 8.6) was 1.1 years greater than that of non-cancer individuals (58.3 ± 7.9). Around half of both groups were males (51.1%, 24/47 of cancer patients and 53.1%, 26/49 of non-cancer individuals). A similar proportion (74.5%, 35/47) of gastric cancer was diagnosed at stage I. The majority (61.7%, 29/47) of gastric tumors were located at distal regions. Three subtypes of gastric cancer by Lauren’s criteria each made up approximately one third of the cancer group. In the non-cancer group, 10.2% (5/49) of the participants had non-cancerous gastric diseases (Table 1; Additional file 2: Table S1).

### Cell-free DNA profiles

We collected a plasma sample from each patient during the initial visit before conducting other screening tests for gastric cancer. Whole genome sequencing was performed to profile cell-free DNA (cfDNA) characteristics. We examined four types of cfDNA mutation and fragmentation-related characteristics (Methods).

**Fig. 2:**
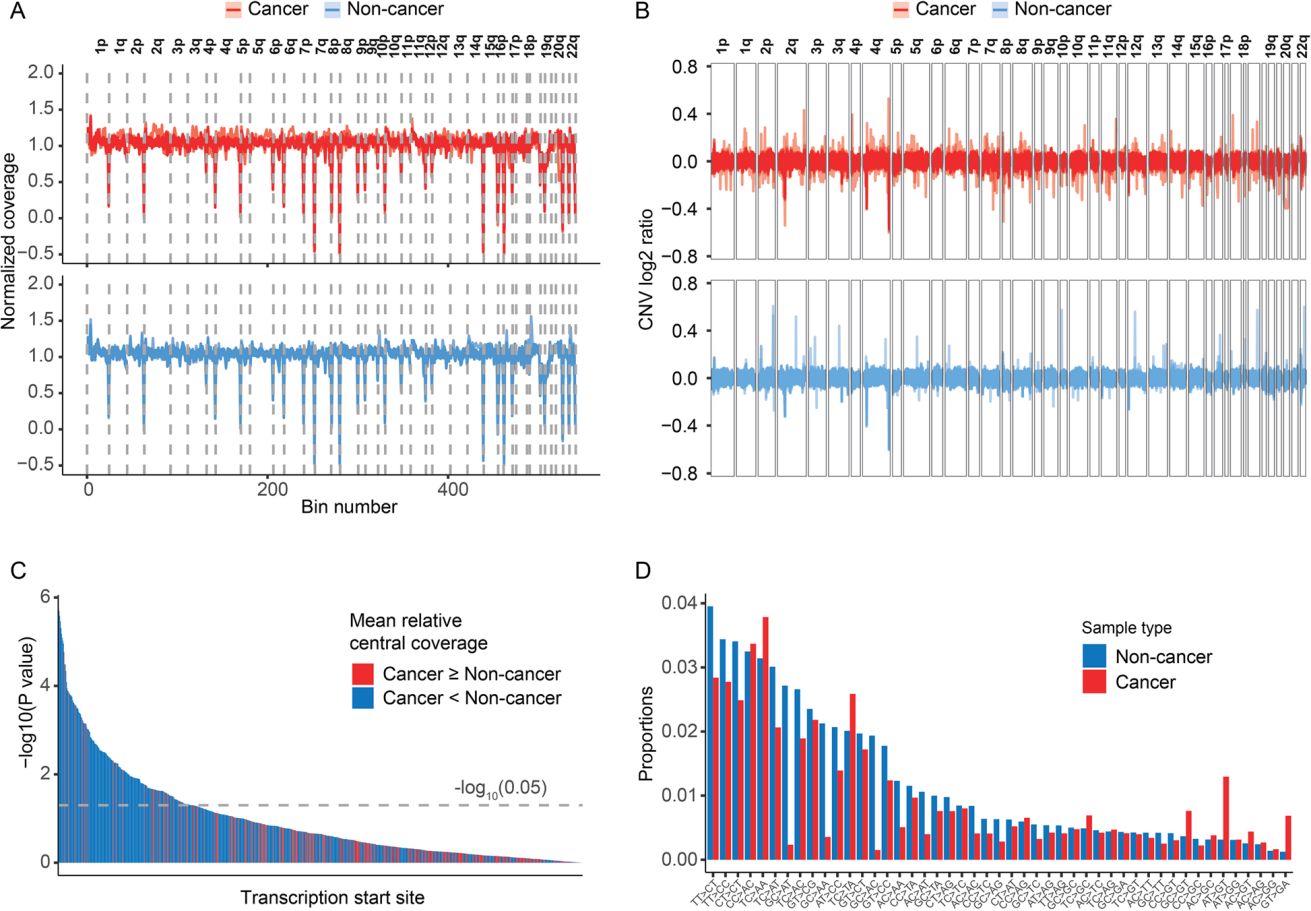
Cell-free DNA profiles. Cell-free DNA characteristics of cancer and non-cancer samples. **A** Normalized long fragment (150–220 bp) coverage values in 541 5-Mb bins over chromosomes of cancer and non-cancer samples. **B** CNV log2 ratio values in 2475 1-Mb bins over chromosomes of cancer and non-cancer samples. **C** *P* values of the comparisons of relative central coverage values between the cancer and non-cancer groups by Wilcoxon tests. Each bar represents the transformed *P* values of a transcription start site. Bars were in a descending order by height. The dotted line denotes the threshold for statistical significance (*P* < 0.05). **D** Mean proportions of each type of single nucleotide substitutions of cancer and non-cancer samples. Single nucleotide substitutions with significant between-group differences in mean proportions were included (*P* < 0.05 by Wilcoxon test). All 110 gastric cancer and 139 non-cancer samples in the study cohort were included in the analyses

The gastric cancer group showed genome-wide variations in the coverage of both long fragments (150–220 bp) and short fragments (100–149 bp). The coverage, however, appeared stable across non-cancer individuals (Fig. 2A; Additional file 1: Fig. S2). In specific regions, such as chromosomal arms 1q, 4q, and 8q [19], gastric cancer patients tended to have greater coverage, which may be contributed, in part, by frequent copy number amplifications (Fig. 2A, B). Diffuse-type gastric carcinomas are associated with more stable genomic profiles, whereas the intestinal type tend to show more copy number alterations [20, 21]. The CNV profiles of intestinal type gastric cancer in our cohort also showed greater variations compared to the diffuse type (Additional file 1: Fig. S3). Coverage at transcription start sites (TSSs) was reported to be negatively associated with respective gene expression levels. We compared the coverages at 854 TSS regions, among which 218 showed significant differences between cancer and non-cancer groups. At most of these regions (89.0%, 194/218), the gastric cancer samples had lower central coverage, indicating higher expression levels. The expressions of genes such as *HMGA1*, *ZNF362*, and *ZNF577*, which are regulated in gastric cancer, were confirmed through RNA-seq data from the oncoDB database [22] (Fig. 2C). The gastric cancer group also exhibited different patterns of mutational signatures. We compared the proportions of 96 types of single-nucleotide substitutions (SNS). 29.2% (28/96) of them had higher proportions in the cancer group (Fig. 2D). These differential cfDNA profiles showed the potential to be applied to distinguishing gastric cancer and non-cancerous conditions.

### Model performance in study and validation cohorts

**Fig. 3:**
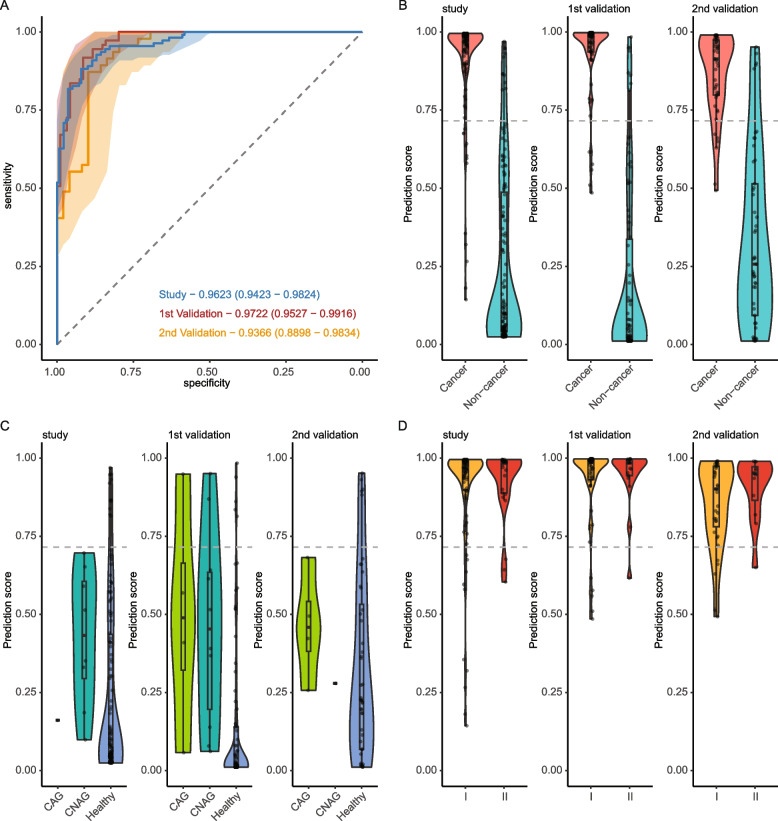
Model performance in study and validation cohorts. Performance of the cfDNA-based assay in distinguishing gastric cancer and non-cancerous conditions in study and validation cohorts. **A** Receiver operating characteristic curves of the model in the study and validation cohorts. **B** Prediction scores of gastric cancer and non-cancer samples in the study (left panel), 1^st^ validation (mid panel), and 2^nd^ validation (right panel) cohorts. **C** Prediction scores of healthy participants and patients with chronic atrophic gastritis and chronic non-atrophic gastritis in the study (left panel), 1^st^ validation (mid panel), and 2^nd^ validation (right panel) cohorts. **D** Prediction scores of gastric cancer patients grouped by pathological stages in the study (left panel), 1^st^ validation (mid panel), and 2^nd^ validation (right panel) cohorts. Dotted horizontal lines denote the threshold at 92.1% specificity in the study cohort. Abbreviations: AUROC, area under receiver operating characteristic curve; CI, confidence interval; CAG, chronic atrophic gastritis; CNAG, chronic non-atrophic gastritis

We developed a machine learning model using these differential cfDNA profiles to distinguish an individual with or without gastric cancer (Methods). The model was fit and tuned with five-fold cross-validations in the study cohort and independently evaluated in the validation cohort (Fig. 1). Using one type of cfDNA feature, the model was able to binarily classify gastric cancer patients and non-cancer individuals with AUROC metrics ranging from 0.841 to 0.912 in the study cohort, from 0.854 to 0.937 in the 1^st^ validation cohort, from 0.802 to 0.900 in the 2^nd^ validation cohort (Additional file 1: Fig. S4; Additional file 2: Table S2). We further combined four types of cfDNA features and constructed an ensemble model. The performance of the ensemble model exceeded those of models with only one feature type. The AUROC metrics reached 0.962 (95% CI: 0.942 – 0.982) in the study cohort, 0.972 (95% CI:0.953 – 0.992) in the 1^st^ validation cohort, and 0.937 (95% CI: 0.890 – 0.983) in the 2^nd^ validation cohort (Fig. 3A). We determined a threshold of 0.715 that maximized the Youden’s index in the study cohort. The ensemble model achieved a specificity of 92.1% (128/139) and a sensitivity of 88.2% (97/110) in the study cohort. With this threshold, we correctly identified 91.5% (86/94) of non-cancer individuals and 91.8% (67/73) of gastric cancer patients in the 1^st^ validation cohort, as well as 89.8% (44/49) of non-cancer individuals and 87.2% (41/47) of gastric cancer patients in the 2^nd^ validation cohorts (Fig. 3B; Additional file 2: Table S3).

All three cohorts included patients with non-cancerous gastric diseases. Their prediction scores were significantly higher than those of healthy individuals (study cohort: 0.398 vs. 0.248, *P* = 0.021; 1^st^ validation cohort: 0.472 vs. 0.152, *P* = 4.863 × 10^−5^; 2^nd^ validation cohort: 0.427 vs. 0.339, *P* = 0.199) (Fig. 3C). Among gastric cancer patients, the prediction scores showed a trend to increase with pathological stages. In the study cohort, the mean prediction scores increased from 0.892 for stage I patients to 0.923 for stage II. The sensitivity was similar between the two groups (stage I: 88.2%, 75/85; stage II: 88.0%, 22/25). In the 1^st^ validation cohort, the mean prediction scores increased from 0.921 for stage I patients to 0.941 for stage II. The sensitivity also increased with the stage (stage I: 91.1%, 51/56; stage II: 94.1%, 16/17). The 2^nd^ validation cohort manifested a stronger increasing trend of prediction scores with pathological stages. The mean prediction scores increased from 0.853 for stage I to 0.906 for stage II. The sensitivity increased from 85.3% (30/35) for stage I and 91.7% (11/12) for stage II (Fig. 3D; Additional file 2: Table S3).

We further examined the prediction scores in other clinicopathological subgroups. The scores did not significantly differ between male and female individuals in both gastric cancer and non-cancer groups across all three cohorts (Additional file 1: Fig. S5, S8A). Gastric cancer of different types by the Lauren classification had similar prediction scores. The mixed type in the study cohort had a slightly lower mean score (0.867) than those of the diffuse and intestinal types (0.910 and 0.907, respectively). The trend was not observed in either validation cohort. In the 1^st^ validation cohort, three subtypes showed almost equal mean scores (0.926, 0.928, and 0.922 for disuse, intestinal, and mixed subtypes, respectively) (Additional file 1: Fig. S6A). In the 2^nd^ validation cohort, the mixed subtype displayed a higher mean score (0.884) than the other two (0.850 and 0.863) (Additional file 1: Fig. S8B). Gastric tumors in proximal locations such as the cardia and fundus were more difficult to detect via conventional gastroscopy than in other locations. Our cfDNA-based assay, however, correctly identified all cases with proximal gastric tumors in all three cohorts (study: 12/12, 1st validation: 7/7, 2^nd^ validation: 6/6) (Additional file 1: Fig. S6B, S8C). Patients with lymph node metastasis tended to have higher mean scores than those without in both validation cohorts (Additional file 1: Fig. S6C, S8D; Additional file 2: Table S3).

To evaluate the representativeness of the validation cohort, we refitted the model with the 1^st^ validation cohort and performed the prediction on the original study cohort reversely. The 1^st^ validation cohort contained 31 samples processed with a different automated liquid handling platform (Methods), which could be one of the possible explanations for their relatively higher prediction scores (Additional file 1: Fig. S9; Additional file 2: Table S1, S4). To avoid the potential influence of Platform 2, we employed only samples processed with Platform 1, comprising 47 gastric cancer and 89 non-cancer samples from the 1^st^ validation cohort, as the training set to refit the model (hereafter denoted as the reverse model). The reverse model showed inferior performance on the testing set (original study cohort) than on the training set (original 1st validation cohort) (AUROC: 0.918 vs. 0.951; sensitivity: 79.1%, 87/110 vs. 85.1%, 40/47; specificity: 87.1%, 121/139 vs. 91.0%, 81/89). This suggested that the training set may be less representative of the testing set or that the sample size of the training set (*N* = 136) may not be large enough for the model to comprehensively learn cancer signal-related cfDNA characteristics. Nonetheless, the reverse model exhibited equivalent classification efficacy in the testing set and 2^nd^ validation cohort (AUROC: 0.918 vs. 0.916; sensitivity: 79.1%, 87/110 vs. 80.9%, 38/47; specificity: 87.1%, 121/139 vs. 85.7%, 42/49), indicating that the 2^nd^ validation cohort may be well representative of the study cohort, especially on gastric cancer samples, for which the pathological stages, lesion locations, and Lauren subtypes were matched (Table 1; Additional file 2: Table S1, S5).

### Model performance in a hypothetical large population

**Fig. 4:**
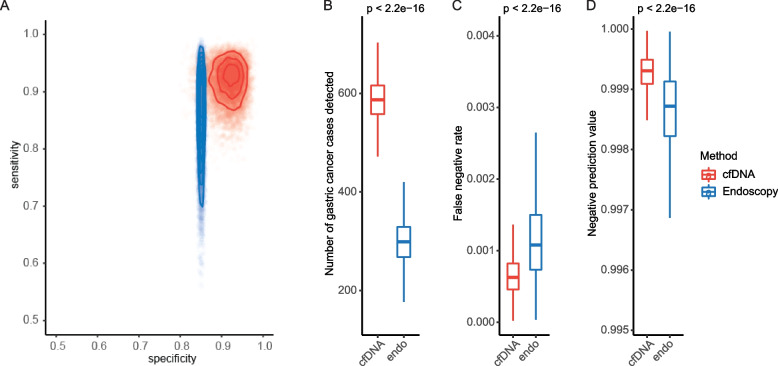
Comparing the cfDNA-based assay and endoscopy in populational gastric cancer screening. In silico comparisons of the cfDNA-based assay and endoscopy screening in a large hypothetical population. **A** Sensitivity and specificity values sampled from prior distributions centered on empirical estimates for 10,000 iterations. **B**–**D** The predictive distributions of true positive incidences (**B**), false negative rates (**C**), and negative prediction values (**D**) of two methods in a hypothetical 100,000 screening population from 10,000 iterations of simulation. The center line in the boxplots represents the median, the upper bound represents the third quantile, and the lower bound represents the first quantile. The upper and lower whisker demotes respective maximum and minimum values of the data that is within 1.5 times the interquartile range. Abbreviation: endo, endoscopy

To evaluate our cfDNA-based assay and conventional gastroscopy at a population scale, we used Monte Carlo simulations to assess the performance metrics in a theoretical population of 100,000 high-risk individuals. We modeled the sensitivity and specificity of our cfDNA-based assay and gastroscopy at prior distributions centered at empirical estimates from our validation cohort and a gastroscopy screening cohort [17], respectively (Fig. 4A). Based on statistics from a multi-center gastric cancer screening trial in China [18], we estimated that the prevalence of gastric cancer in the theoretical population would be 0.8% and that the overall compliance rate for gastroscopy would be 43.8%. Considering previous reports that blood tests typically achieve adherence rates of 80–90% [23, 24] and our experience in clinical practice, we conservatively assumed that an average of 80% of the screening population would complete our tests. With these priors, the Monte Carlo simulations revealed that gastroscopy may on average detect 299 (95% CI: 268–334) gastric cancer cases. Our cfDNA-based assay could potentially identify 587 (95% CI: 542–634) cases, almost twice as many as the number by gastroscopy (Fig. 4B). The false negative rate meanwhile could also be nearly halved with our approach (0.066%) compared to gastroscopy (0.116%) (Fig. 4C). Additionally, our assay could also improve the accuracy of negative testing results — increasing the negative prediction values from an average of 99.86% to 99.93% (Fig. 4D). These results suggest that our cfDNA-based assay may provide population-wide benefits for gastric cancer screening. Nonetheless, it is important to note that the conclusions drawn from the in silico comparisons between our cfDNA-based assay and gastroscopy are preliminary and of very limited clinical significance. Large heterogenous prospective cohorts in the real world are required for systemic comparisons of novel and conventional methods.

## Discussion

In this study, we introduced a cfDNA-based liquid biopsy assay capable of accurately identifying patients with early-stage gastric cancer from non-cancer individuals. Both the study and validation cohorts included only early-stage (stage I–II) gastric cancer patients, which are typically challenging for conventional screening modalities to detect. Despite this, our assay achieved an AUROC of 0.962 (95% CI: 0.942–0.982) in the study cohort, 0.972 (95% CI:0.953–0.992) in the first validation cohort, and 0.937 (95% CI: 0.890–0.983) in the second validation cohort.

Our assay utilized four types of cfDNA features, none of which has been tested in other gastric cancer studies focused on early detection. We showed that all four types of features embody signals of circulating tumor DNA. Beyond visualizing differences between groups, we developed a machine learning model to estimate the probability of cancer presence. Samples from gastric cancer patients of different pathological stages (stage I or II, N stage 0 or +) and Lauren classifications (diffuse or intestinal type), and non-cancer individuals of different conditions (with or without gastric disease) exhibited distinct cancer probability levels (Additional file 1: Fig. S5–S6, S8). These results suggest that our method may provide a reliable quantification of gastric cancer signals.

Our cohorts included only early-stage gastric cancer, some of which were located at the cardia and fundus regions. These tumors were relatively less prevalent in real-world populations and often could be of challenge for conventional gastroscopy to detect at early stages. Nonetheless, our cfDNA-based assay achieved high sensitivity regardless of tumor locations. Of note, in all three cohorts, all gastric tumors at proximal locations (25/25) were correctly identified by our approach. Considering the non-invasiveness, accessibility, and cost-effectiveness, it is likely that the compliance rate for our cfDNA-based approach could be significantly higher than other conventional modalities in the real-world screening population. Through in silico simulations based on presumptions of ideal conditions, we estimated that our approach may have the potential to detect ~ 300 more gastric cancer cases than gastroscopy alone per 100,000 high-risk individuals screened, which could be largely due to the advantages in patient compliance and assay robustness across tumor locations. However, we must emphasize that these estimations from virtual simulations are preliminary and have limited clinical impact without real-world validations in a practice setting whereby compliance, sensitivity, and specificity could be rigorously assessed.

Our cohorts also included 23 participants with chronic gastritis, with 5 atrophic and 18 non-atrophic forms. In both validation cohorts, the chronic atrophic gastritis (CAG) group had a higher mean score (0.496 and 0.464) than that (0.463 and 0.279) of the chronic non-atrophic gastritis (CNAG) group, though lacking statistical significance due to the small sample size (Additional file 2: Table S1).

Previous studies have evaluated cfDNA level as a biomarker for detecting gastric cancer, assessing treatment responses, and/or predicting the prognosis. Gastric cancer patients receiving different therapeutic modalities, such as curative resection [25], chemotherapy [26–28], targeted therapy [27], and immunotherapy [29], were enrolled in these studies. The quantitative indicators for cfDNA levels also diverged from the cfDNA concentration [25, 28], the *Alu247/Alu115* ratio [26, 30], and the *Alu81* copy number [31], to the total cfDNA copy number from qPCR [27]. Our cfDNA-based assay was primarily designed for the early detection of gastric cancer. Therefore, our cohorts enrolled only stage I–II patients without prior cancer treatment, who were more likely to benefit from real-world population screenings. We employed multi-dimensional cfDNA characteristics profiled from low-coverage whole-genome sequencing data to identify these early-stage gastric cancer patients from non-cancer individuals [32]. Compared to the sole indicators of cfDNA levels in previous studies, the depth and breadth of our cfDNA features allowed for sensitive and stable detection of gastric cancer signals. Moreover, the combination of multiple dimensions of cfDNA profiles could help mitigate the effects of potential signal shifts and background noise. Our validation cohort, which was separately collected in a different time frame further validated the utility of the multi-dimensional cfDNA characteristics and model framework in the early detection of gastric cancer. The sample sizes of both study and validation cohorts also enhanced the reliability of our assay.

This study is not without limitations. First, our cohorts consisted of only Asian participants. As differential genomic alterations have been known among populations, the generalizability of our model needs to be further investigated in larger and more diverse cohorts. Second, the 1^st^validation cohort may be less representative of the study cohort and may contain potential batch effects introduced by different automated liquid handling platforms. Third, our in silico simulation was based on several presumptions of ideal conditions, which may differ in real-world screening scenarios. The relatively small sample size and lack of populational heterogeneity also impact the clinical significance of the simulation. Fourth, fragmentomic features used in our assay may lack sufficient biological explanations for individual variables (Additional file 1: Fig. S7). Although multiple studies have reported the association of fragmentomic patterns and epigenetic regulations [3], their biological significance for gastric cancer detection remains not fully understood. Finally, we are also unable to provide an objective comparison with other emerging liquid biopsy methods such as methylation-based inference due to the lack of methylation data.

For the 31 samples in the 1^st^ validation cohort processed with Platform 2, despite showing relatively higher prediction scores, 26 gastric cancer and 5 non-cancer samples in this set remained well differentiated, achieving an AUROC of 0.969 (95% CI: 0.903–1.000). At the predefined threshold from the study cohort, this group still demonstrated a high sensitivity of 100.0% (26/26) and a moderate specificity of 80.0% (4/5). Given the clear differentiation and small sample size of this set, we cannot rule out stochastic effects from the underlying factors for higher prediction scores. The potential association of Platform 2 with score elevation remains to be further investigated. Our medical diagnostic laboratory currently utilizes both automated liquid handling platforms. By gathering more samples from both platforms in parallel, we will be able to comprehensively assess the influence of different liquid handling operations on our assay. Should dataset shifts occur due to a change in platform, we plan to develop domain adaptation algorithms to ensure our assay performs effectively and consistently across different platforms.

In summary, we have provided evidence that multi-dimensional cfDNA profiles can accurately identify gastric cancer. Our assay yielded stable and robust performance in both a temporally separated validation cohort and an independent validation cohort.

## Conclusions

In this study, we introduced a liquid biopsy assay employing four types of cfDNA profiles to accurately distinguish early-stage gastric cancer from non-cancerous conditions. The assay demonstrated robust performance across various subgroups, including different pathological stages, Lauren classifications, and tumor locations. Its efficacy was validated in both internal and external cohorts.

### Supplementary Information

Additional file 1. Supplementary figures including Fig. S1-S9 in the .pdf format.

Additional file 2. Supplementary tables including Table S1-S5 in the .xlsx format.

Additional file 3. Descriptions of supplementary tables in the .pdf format.

Additional file 4. Study protocol in the .pdf format.

## Data Availability

The original data presented in the study have been deposited in the Genome Sequence Archive for Human (GSA-Human) repository under the identifier (HRA005926) (https://ngdc.cncb.ac.cn/gsa-human/browse/HRA005926) [32].
