# Supplementary material for: Multi-dimensional cell-free DNA-based liquid biopsy for sensitive early detection of gastric cancer
Source: Genome Med. 2024 Jun 7;16:79. doi: 10.1186/s13073-024-01352-1 (PMC11157707; doi:10.1186/s13073-024-01352-1)

Legends for supplementary figures

Figure S1. Cohort inclusion and exclusion

Flowchart illustrating the inclusion and exclusion processes.

Abbreviations: QC, quality control; CT, computed tomography; CEA, carcinoembryonic antigen; CA19-9, arbohydrate antigen 19-9; CA125, carbohydrate antigen 125; PSA, prostate specific antigen; AFP, alpha-fetoprotein.

Figure S2. Cell-free DNA fragment size pattern profiles

Normalized shot fragment (100 - 149bp) coverage values in 541 5Mb-bins over chromosomes of cancer and non-cancer samples. All 110 gastric cancer and 139 non-cancer samples in the study cohort were included.

Figure S3. CNV profiles of participants grouped by Lauren's classification

CNV log2 ratio values in 2475 1Mb-bins over chromosomes of the diffuse type, intestinal type, mixed type, and non-cancer groups.

Abbreviations: CNV - copy number variation.

Figure S4. Model performance by single feature type

Performance of the model using each type of cfDNA profile in distinguishing gastric cancer and non-cancerous conditions in the study and validation cohorts.

Abbreviation: CNV - copy number variation, FSP - fragment size pattern, NCP - nucleosome coverage pattern, SNS - single nucleotide substitution.

Figure S5. Prediction scores of samples grouped by sex

Prediction scores of gastric cancer and non-cancer samples grouped by sex in the study (upper panel) and validation (lower panel) cohorts. Dotted horizontal lines denote the threshold at 92.1% specificity in the study cohort.

Figure S6. Prediction scores of gastric cancer samples grouped by Lauren classification, location, and N stage

A-C). Prediction scores of gastric cancer samples grouped by Lauren classification (A), location (B), and N stage (C) in the study (left panel) and validation (right panel) cohorts. Dotted horizontal lines denote the threshold at 92.1% specificity in the study cohort.

Figure S7. Variable importance of base and ensemble models

A). Relative importance of 20 base models (5 base model per feature type). B-E). Twenty variables of the highest relative importance in each feature type.

Figure S8. Prediction scores of the 2nd validation cohort in subgroups

A). Prediction scores of gastric cancer and non-cancer samples in the 2nd validation grouped by sex. B-D). Prediction scores of gastric cancer samples in the 2nd validation grouped by Lauren subtype (B), tumor location (C), and N stage (D).

Figure S9. Prediction scores with platform labeled

Prediction scores of three cohorts. Colors denote the types of automated liquid handling platforms.

Figure S1. Cohort inclusion and exclusion

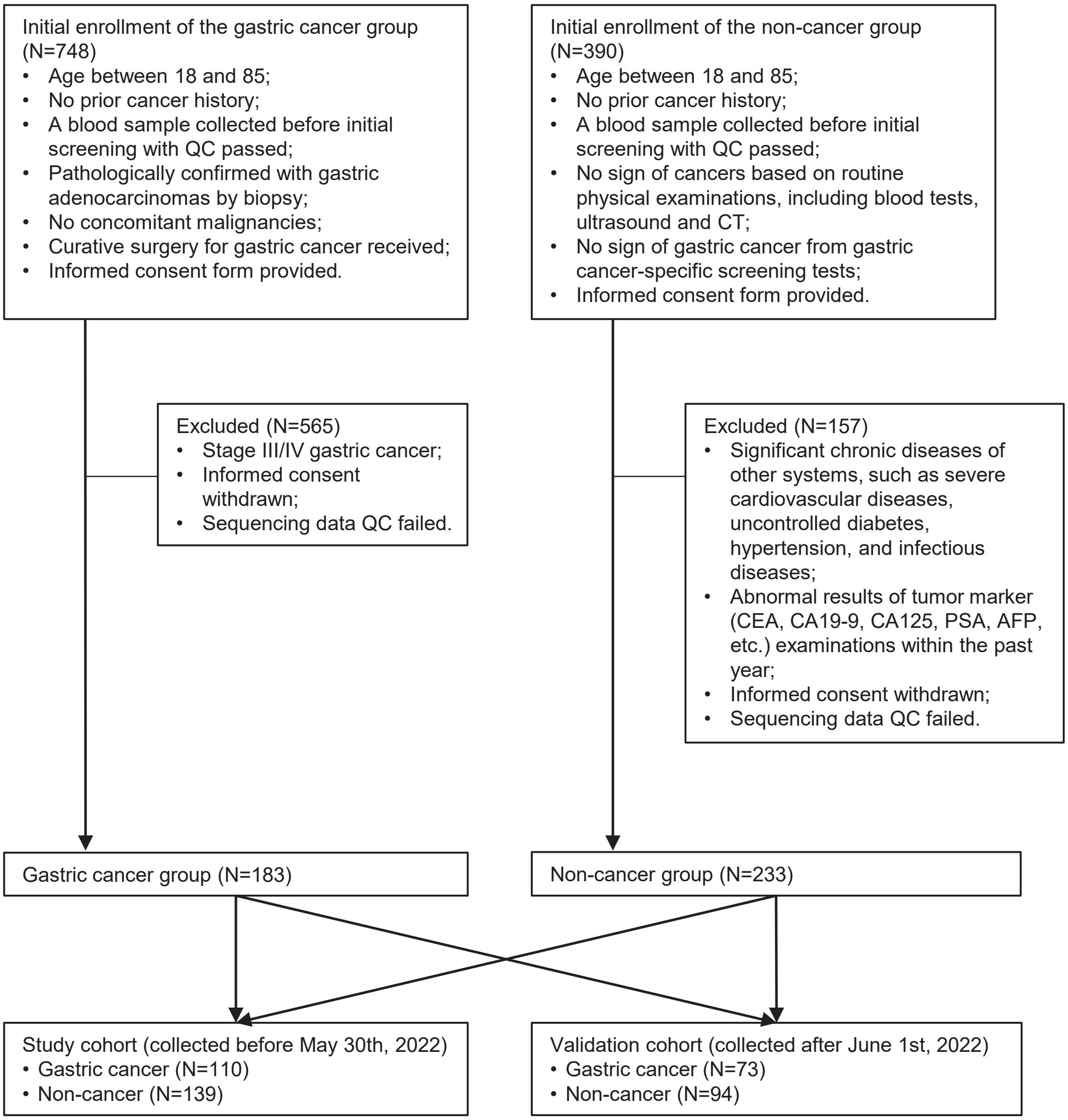

Figure S2. Cell-free DNA fragment size pattern profiles

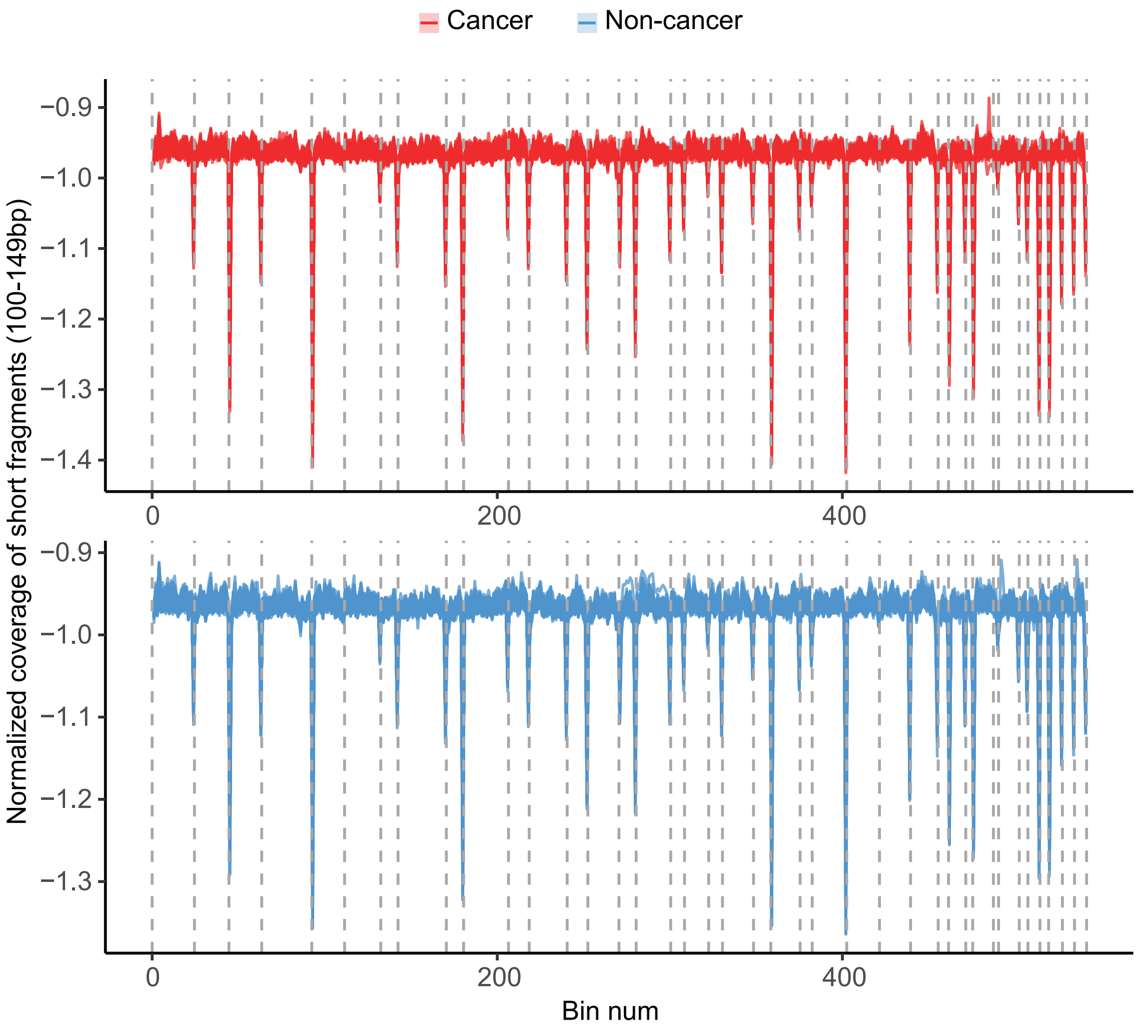

Figure S3. CNV profiles of participants grouped by Lauren's classification

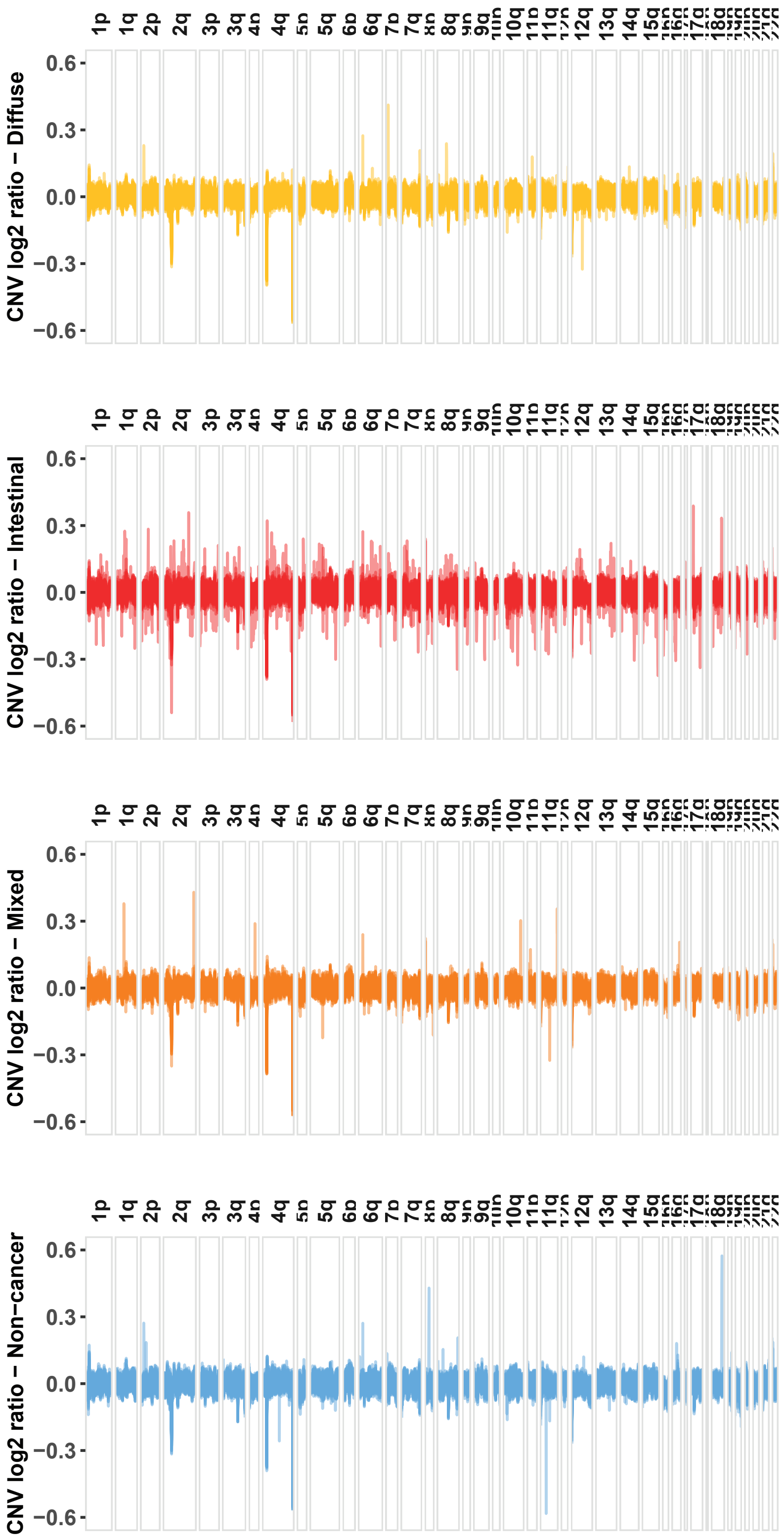

Figure S4. Model performance by single feature type

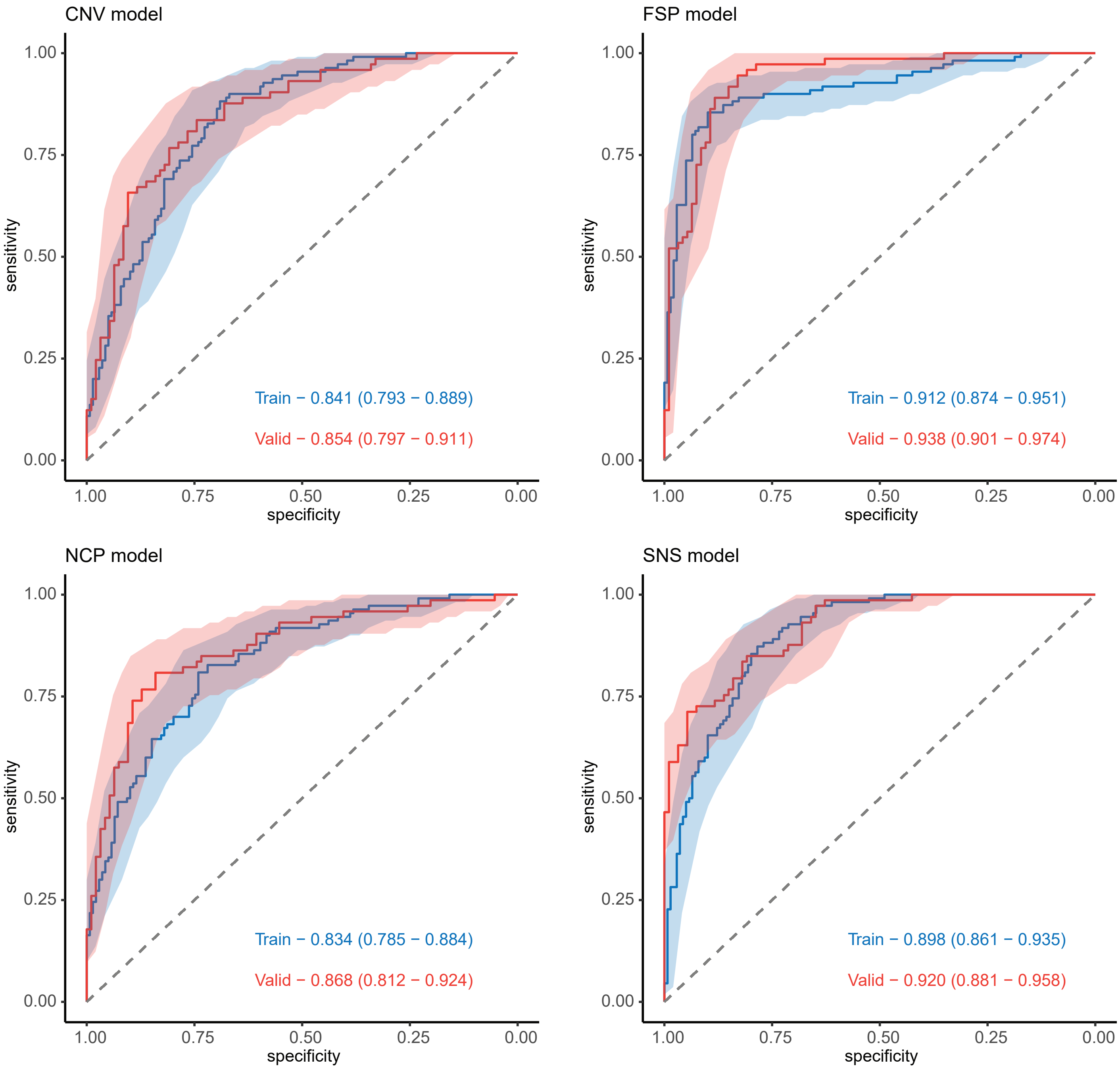

Figure S5. Prediction scores of samples grouped by sex

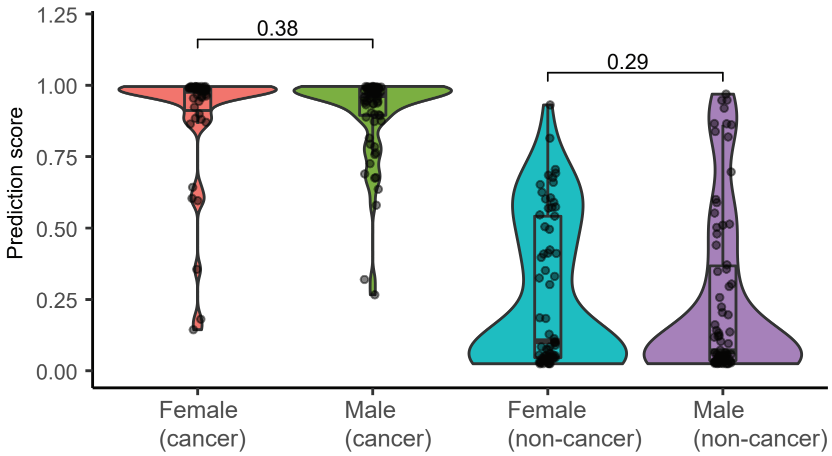

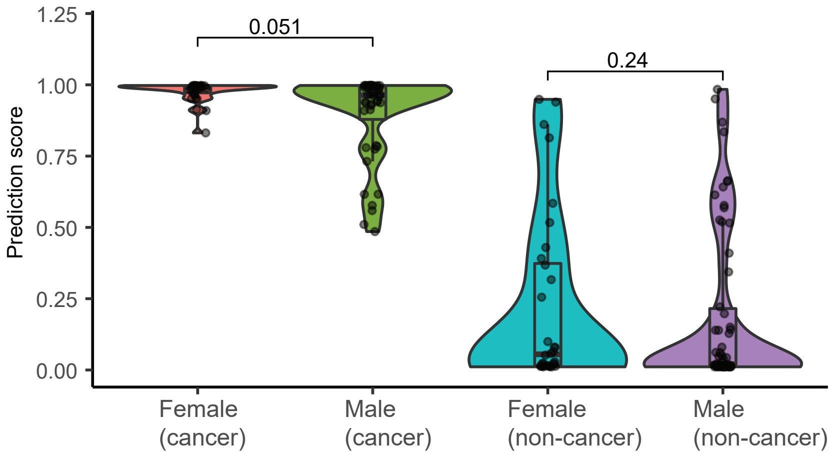

Figure S6. Prediction scores of gastric cancer samples grouped by Lauren classification, location, and N stage

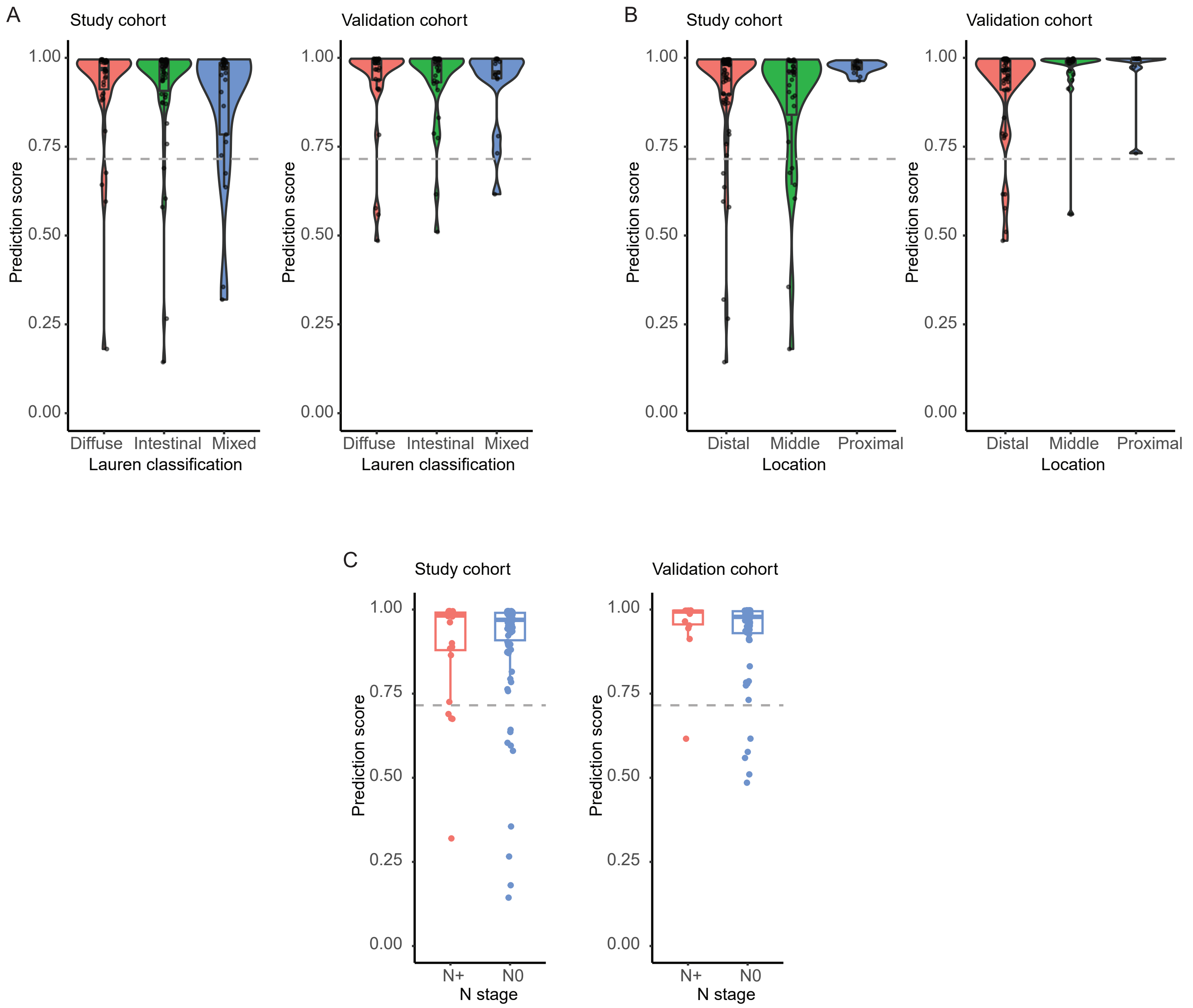

Figure S7. Variable importance of base and ensemble models

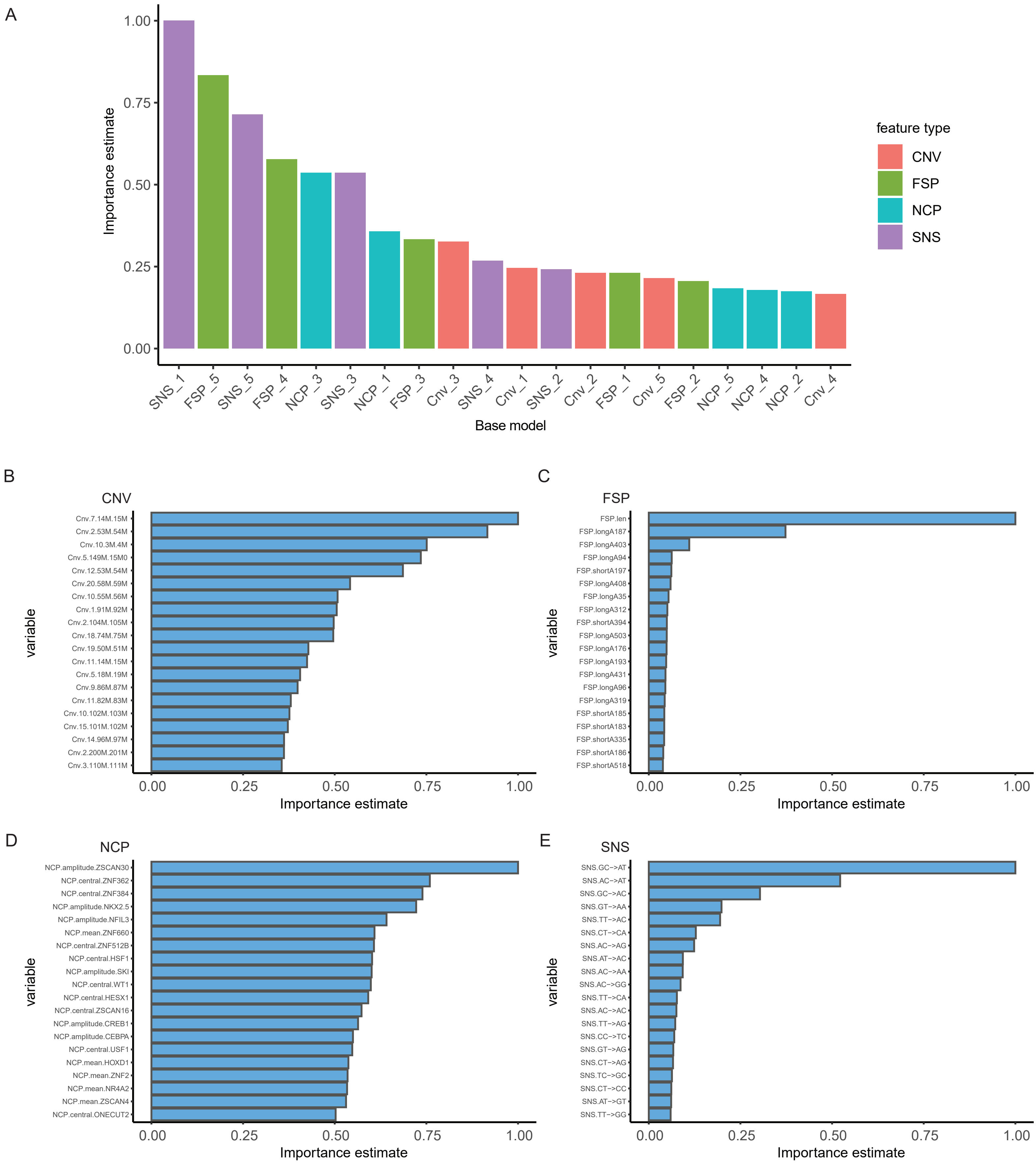

Figure S8. Prediction scores of the 2nd validation cohort in subgroups

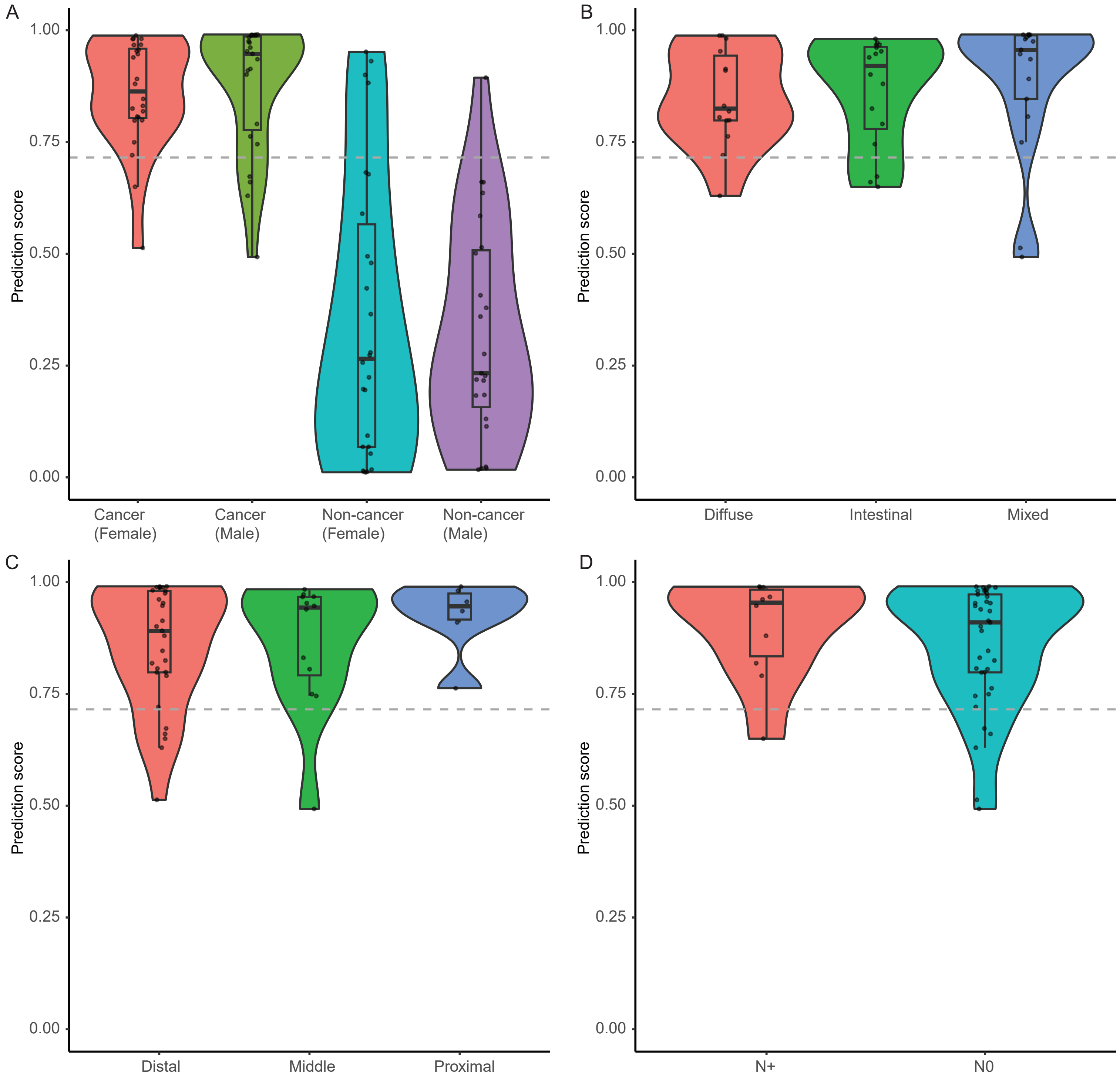

Figure S9. Prediction scores with platform labeled

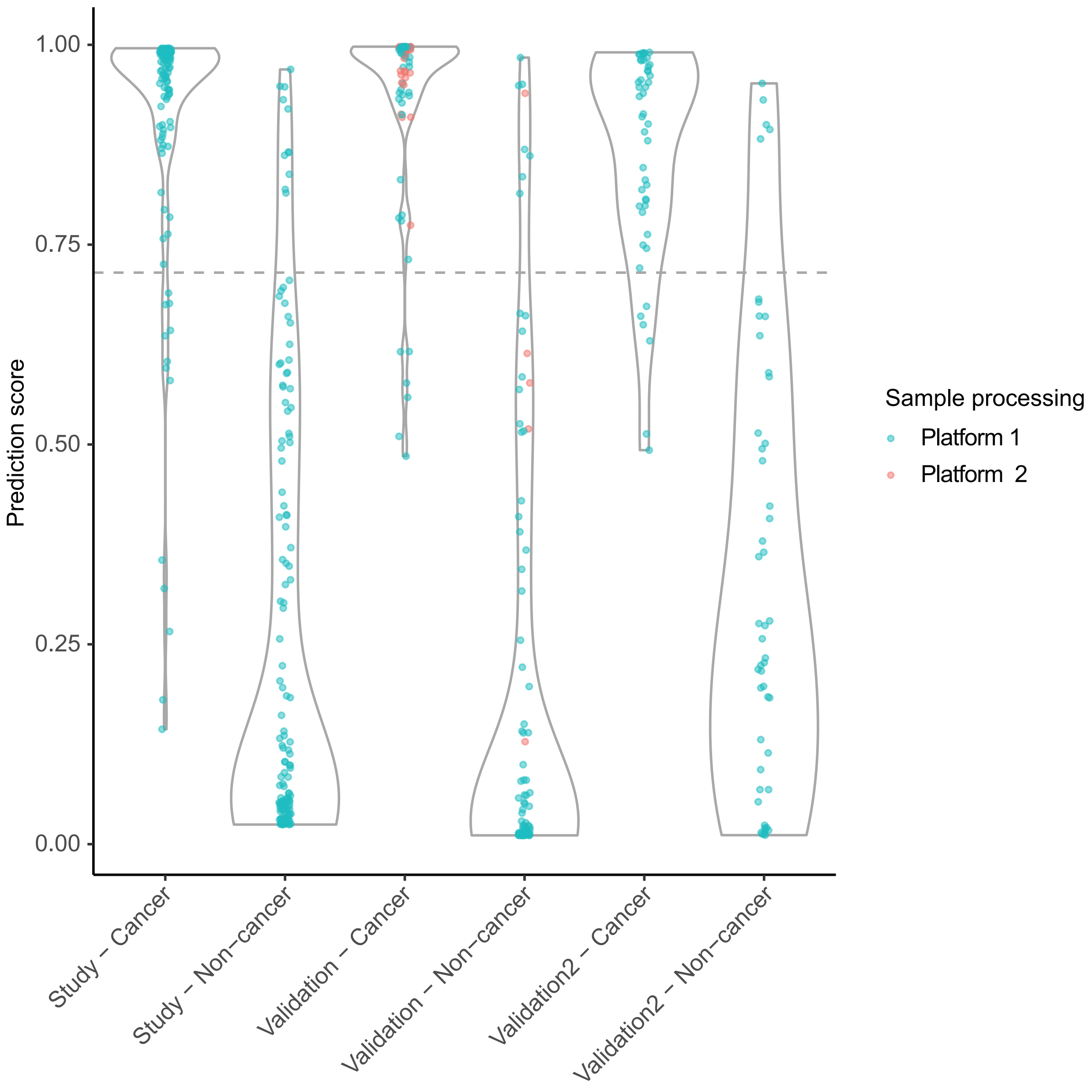

Supplement: Supplementary file 1 — Additional file 1. Supplementary figures including Fig. S1-S9 in the .pdf format. [file 13073_2024_1352_MOESM1_ESM.pdf]
