# Supplementary material for: Multi-dimensional cell-free DNA-based liquid biopsy for sensitive early detection of gastric cancer
Source: Genome Med. 2024 Jun 7;16:79. doi: 10.1186/s13073-024-01352-1 (PMC11157707; doi:10.1186/s13073-024-01352-1)
Supplement: Supplementary file 3 — Additional file 3. Descriptions of supplementary tables in the .pdf format. [file 13073_2024_1352_MOESM3_ESM.docx]

**Descriptions of supplementary tables**

**Table S1. Clinicopathological information of participants**

Clinicopathological information and prediction scores of each participant in three cohorts.

**Table S2. Performance metrics of single-feature and ensemble models**

AUROC metrics of singe-feature models and the ensemble model in three cohorts.

Abbreviations: AUROC - area under receiver-operating characteristic curve; CI - confidence interval; CNV - copy number variation; FSP - fragment size pattern; NCP - nucleosome coverage pattern; SNS - single nucleotide substitution.

**Table S3. Performance metrics in clinicopathological subgroups**

Performance metrics of the ensemble model in samples grouped by clinicopathological information in three cohorts.

Abbreviations: AUROC - area under receiver-operating characteristic curve; CI - confidence interval

**Table S4. Performance metrics in two platforms**

Performance metrics of the ensemble model in samples grouped by automated liquid handling platform type in three cohorts.

Abbreviations: AUROC - area under receiver-operating characteristic curve; CI - confidence interval

**Table S5. Performance metrics of the reverse model**

Performance metrics of the reverse model in samples grouped by automated liquid handling platform type in three cohorts.

Abbreviations: AUROC - area under receiver-operating characteristic curve; CI - confidence interval
