# Supplementary material for: Multi-dimensional cell-free DNA-based liquid biopsy for sensitive early detection of gastric cancer
Source: Genome Med. 2024 Jun 7;16:79. doi: 10.1186/s13073-024-01352-1 (PMC11157707; doi:10.1186/s13073-024-01352-1)
Supplement: Supplementary file 4 — Additional file 4. Study protocol in the .pdf format. [file 13073_2024_1352_MOESM4_ESM.pdf]

Study Protocol

Study Title	Multi-dimensional cell-free DNA-based liquid biopsy for sensitive early detection of gastric cancer
Number of Sites	Single site
Rationale	Gastric cancer remains as one of the leading causes of death worldwide. Detecting gastric cancer earlier is vital for managing the disease effectively, improvement of survival rates and reduction of morbidity. Blood-based liquid biopsy has emerged as a promising technology for the early detection of multiple types of cancer, which can complement traditional cancer screening methods in a non-invasive manner. We developed a novel liquid biopsy assay using multi-dimensional cell-free DNA (cfDNA) features from whole genomic sequencing (WGS) data for the detection of gastric cancer at early stages.
Study Design	A single-center prospective observational study will be used to develop and valid the gastric cancer early detection study. The study is divided into two phases. The first phase of the study will enroll ~250 participants, including ~100 stage I-II gastric cancer patients and ~150 non-cancer individuals, over a period of 8 months. The cfDNA profiles of these participants will be used to develop a multi-layer machine learning model for identifying cancer presences. The second phase of the study will enroll ~125 participants over a period of 4 months to validate the performance of the cfDNA-based assay.
Primary Outcomes	The area under the receiver operator characteristic curve (AUROC) metric for differentiating stage I/II gastric cancer patients and non-cancer individuals using the cfDNA-based assay.
Secondary Outcomes	The sensitivity and specificity for differentiating stage I/II gastric cancer patients and non-cancer individuals using the cfDNA-based assay.
Interventions	Not applicable
Number of Samples	First phase: ~100 stage I-II gastric cancer patients, ~150 non-cancer individuals.

	Second phase: ~50 stage I-II gastric cancer patients, ~75 non-cancer individuals.
Procedure	This prospective study collects blood samples from participants with a new diagnosis of cancer (cancer arm) and from participants who do not have a diagnosis of cancer (non-cancer arm) to develop and validate the cfDNA-based assay for the early detection of gastric cancer.
Inclusion Criteria	Inclusion criteria for all the participants:<br>• 18 – 85 years old.<br>• Individuals without a prior history of cancer.<br>• Individuals with a plasma sample that was collected before initial screening tests and passed quality control (QC).<br>• Individuals who provided informed consent.<br>Inclusion criteria for cancer arm participants:<br>• Individuals who were pathologically confirmed with gastric adenocarcinomas by biopsies without concomitant malignancies and received curative surgeries for gastric cancer.<br>Inclusion criteria for non-cancer arm participants:<br>• Individuals who showed no signs of cancers based on routine physical examinations, including blood tests, ultrasound and CT, and no sign of gastric cancer from gastric cancer-specific screening tests.
Exclusion Criteria	Exclusion criteria for all the participants:<br>• Individuals who withdrew informed consent.<br>• Individuals whose sequencing data failed QC.<br>Exclusion criteria for cancer arm participants:<br>• Individuals who were pathologically confirmed with stage III/IV gastric cancer.<br>Exclusion criteria for non-cancer arm participants:<br>• Individuals with significant chronic diseases of other systems, such as severe cardiovascular diseases, uncontrolled diabetes, hypertension, and infectious diseases.

	• Individuals who had abnormal results of tumor marker (CEA, CA19-9, CA125, PSA, AFP, etc.) examinations within the past year.
Sample Enrollment	The study will enroll participants who voluntarily register in this clinical study at Zhejiang Cancer Hospital. All participants will be pre-screened as per Inclusion and Exclusion Criteria.
Test	Blood samples will be collected and processed at Zhejiang Cancer Hospital.
Statistical Analysis	The comparison of continuous numeric data will be conducted using the Wilcoxon test. The comparison of proportions between groups will be conducted using the Fisher's exact test. The trend of continuous numeric data across ordered groups will be assessed using Jonckheere trend test. A two-sided P value of less than 0.05 will be considered significant for all tests unless otherwise indicated.
Overall Duration	Estimated about 12 months

1 INTRODUCTION

1.1 Introduction

Gastric cancer is one of the major global health challenges and leading causes of death. Worldwide, it is estimated that 1,089,103 people were diagnosed with gastric cancer and 768,793 deaths from gastric cancer occurred in 2020(1). Unfortunately, 62% of gastric cancers are detected after the cancer has already spread, either regionally or distantly, resulting in limited treatment options and poor prognosis. Patients with advanced-stage gastric cancer have an extremely low 5-year survival rate of 6%. However, if gastric cancer is diagnosed and treated at early stages before dissemination, the 5-year survival rate is 72%(1). Therefore, there is a need for optimized tools to detect gastric cancer earlier in order to manage the disease effectively, improve survival rates, and reduce morbidity.

Currently, there are a few screening tests available for gastric cancer, such as gastroscopy, computed tomography, Barium Swallow X-ray, *Helicobacter pylori* Antibody Test etc. However, these conventional methods are limited by high cost, invasiveness, poor compliance, or

relatively low accuracy. A cost-effective, non-invasive, and accurate assay is highly desired for regular screening for gastric cancer.

Cell-free DNA (cfDNA)-based liquid biopsy has emerged as a promising technology for the early detection of gastric cancer, offering a non-invasive complement to traditional cancer screening methods. Unlike conventional screening and tissue biopsy, cfDNA-based liquid biopsy only requires a blood sample, making the process quicker and more convenient. cfDNA consists of fragmented genomic DNA released into the bloodstream through mechanisms such as apoptosis, necrosis, and secretion. Tumor-derived cfDNA, known as circulating tumor DNA (ctDNA), carries genetic and epigenetic aberrations characteristic of cancer, including mutations, copy number alterations, aberrant methylation, and abnormal fragmentation. Through next-generation sequencing and computational algorithms, ctDNA can be distinguished from the total pool of cfDNA, enabling the accurate early detection of gastric cancer.

We have preliminarily assessed the efficacy of various cfDNA characteristics, including fragment size pattern (FSP), copy number variation (CNV), nucleosome coverage pattern (NCP), and single nucleotide substitutions (SNS), in detecting gastric cancer signals. In this study, our goal is to develop a cfDNA-based liquid biopsy assay that leverages multi-dimensional cfDNA features profiled from low-depth whole-genome sequencing data to accurately identify gastric cancer at early stages.

1.2 Purpose

1st Phase – Assay development and preliminary validation:

The objective of the first phase is to design, develop, and internally validate a cfDNA-based liquid biopsy assay optimized for the precision of identifying early-stage gastric cancer patients from non-cancer individuals. This phase also aims to determine a clinically meaningful threshold that effectively balances sensitivity and specificity.

2nd Phase – Assay validation:

The objective of the second phase is to collect samples to independently validate the performance of the assay developed in the first phase.

2 STUDY OBJECTIVES

2.1 Primary Objectives (Primary Outcomes)

1st Phase – Assay development and preliminary validation:

- Utilize the data collected in this phase to comprehensively profile cfDNA characteristics.
- Develop a classification model to distinguish gastric cancer patients from non-cancer individuals.
- Conduct five-fold cross validations to assess the assay's efficacy and calculate the AUROC metric for binary classification.

2nd Phase – Assay validation:

- Use the assay developed in the 1st phase to predict the cancer presences in the participants enrolled in this phase and calculate the AUROC metric for the predictions.

2.2 Secondary Objectives (Secondary Outcomes)

1st Phase – Assay development and preliminary validation:

- Establish an appropriate threshold that maximize Youden's index from cross validations.
- Calculate the sensitivity and specificity of the assay at this threshold.

2nd Phase – Assay validation:

- Calculate the sensitivity and specificity of the assay at the predefined threshold from the 1st phase.

3 STUDY DESIGN

3.1 CLINICAL TRAIL FLOW CHART

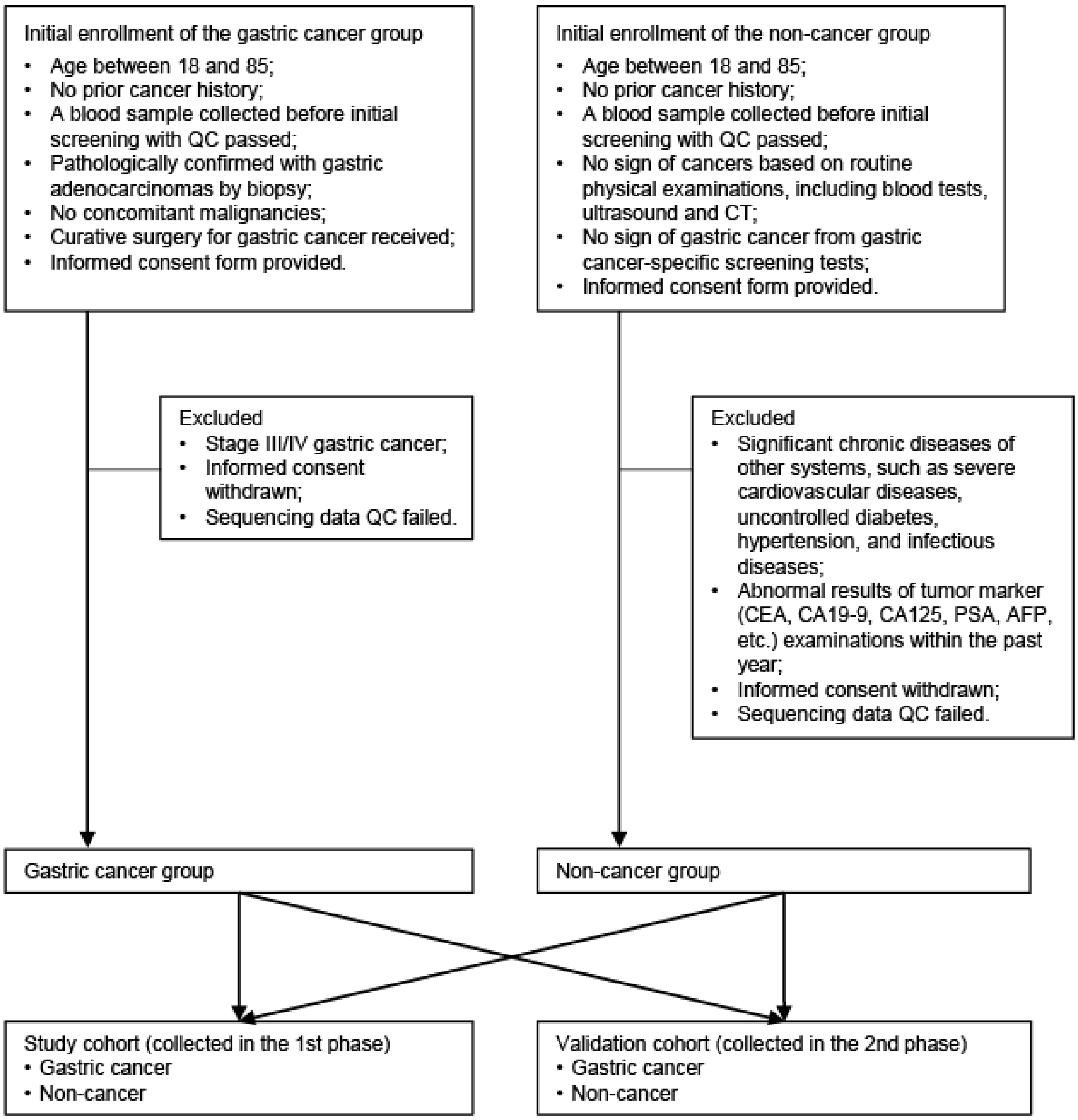

4 SPONSOR

Not applicable

5 POPULATION

5.1 Inclusion Criteria

Inclusion criteria for all the participants:

- 18 – 85 years old.
- Individuals without a prior history of cancer.
- Individuals with a plasma sample that was collected before initial screening tests and passed quality control (QC).
- Individuals who provided informed consent.

Inclusion criteria for cancer arm participants:

- Individuals who were pathologically confirmed with gastric adenocarcinomas by biopsies without concomitant malignancies and received curative surgeries for gastric cancer.

Inclusion criteria for non-cancer arm participants:

- Individuals who showed no signs of cancers based on routine physical examinations, including blood tests, ultrasound and CT, and no sign of gastric cancer from gastric cancer-specific screening tests.

5.2 Exclusion Criteria

Exclusion criteria for all the participants:

- Individuals who withdrew informed consent.
- Individuals whose sequencing data failed QC.

Exclusion criteria for cancer arm participants:

- Individuals who were pathologically confirmed with stage III/IV gastric cancer.

Exclusion criteria for non-cancer arm participants:

- Individuals with significant chronic diseases of other systems, such as severe cardiovascular diseases, uncontrolled diabetes, hypertension, and infectious diseases.
- Individuals who had abnormal results of tumor marker (CEA, CA19-9, CA125, PSA, AFP, etc.) examinations within the past year.

6 BASELINE CHARACTERISTICS

The participants will be adults of 18-85 years with no prior cancer history. The cancer arm will include patients diagnosed with stage I/II gastric cancer. The non-cancer arm will consist of individuals with no known or suspected cancer diagnosis.

7 TREATMENT

Not applicable

8 ENROLLMENT

7.1 Participant recruitment:

Participants will be sourced from Zhejiang Cancer Hospital.

7.2 Preliminary assessment and blood sample collection:

After expressing interest or being approached for the study, participants will undergo a thorough assessment based on a predefined set of Inclusion and Exclusion criteria. This assessment ensures that only those individuals who meet the study's specific requirements and standards will be enrolled. A blood sample from each eligible participant will be collected following standard procedures.

7.3 Categorization:

After enrollment, participants will be categorized into two main arms based on their medical status:

Cancer arm: participants diagnosed with gastric cancer.

Non-cancer arm: participants with no known or suspected cancer diagnosis.

7.4 Sample processing:

Plasma will be isolated from blood samples within 4 hours of collection and then stored at 4℃. cfDNA will be extracted from plasma within 72 hours and stored at -80℃ for later whole-genome sequencing (WGS). cfDNA profiles will be generated from WGS data using in-house analyzing pipelines.

9 ADVERSE EFFECT REPORTING

The in-vitro assay is in the same form as regular blood tests during physical examinations. Participant will not be informed of the analysis results. We expect no additional adverse effects specifically associated with this liquid biopsy assay. Routine adverse effects reporting procedures will apply.

10 STATISTICAL ANALYSIS

The comparison of continuous numeric data will be conducted using the Wilcoxon test. The comparison of proportions between groups will be conducted using the Fisher's exact test. The trend of continuous numeric data across ordered groups will be assessed using Jonckheere trend test. A two-sided P value of less than 0.05 will be considered significant for all tests unless otherwise indicated.

11. ETHICS AND REGULATORY REQUIREMENTS

Written consent will be obtained from the participants from whom the samples are collected before the time of sample collection. The test requisition forms, records, and results will be kept in a confidential state and not be disclosed to any participants or third-party organizations/ individuals. All testing results will be only used for this research study. No testing result will be used for diagnostic or prescriptive purposes. All conducted procedures will be in compliance with the Declaration of Helsinki.

References

1. Siegel RL, Miller KD, Fuchs HE, Jemal A. Cancer statistics, 2022. CA Cancer J Clin. 2022;72(1):7-33.
